# Bimetallic Cu/Fe-MOF-based heterojunction sonozymes for triple amplification of sono-immunotherapy through activating tumor-specific cuproptosis and cGAS-STING pathway

**DOI:** 10.7150/thno.130381

**Published:** 2026-02-26

**Authors:** Xueyuan Liu, Nan Wang, Jinming Cai, Jinyan Hu, Zhenlin Zhang, Chuanqi Feng, Dengyu Pan, Bijiang Geng

**Affiliations:** 1 School of Environmental and Chemical Engineering, Shanghai University, Shanghai 200444, China.; 2 Senior Department of Obstetrics and Gynecology, Chinese PLA General Hospital, Beijing 100007, China.; 3 Department of Obstetrics and Gynecology, the First Medical Center, Chinese PLA General Hospital, Beijing 100853, China.; 4 Shandong Provincial Key Laboratory of Monocrystalline Silicon Semiconductor Materials and Technology, College of Chemistry and Chemical Engineering, Dezhou University, Dezhou, Shandong 253023, China.

**Keywords:** Fe-based metal-organic frameworks, Cu doping, oxygen-vacancy-doped MnO_2-x_, cGAS-STING activation, sono-immunotherapy

## Abstract

**Background:**

Initiating ROS-induced ICD and activating innate immune pathways are promising strategies for reprogramming immunosuppressive TME and eliciting persistent antitumor immune responses.

**Methods:**

To realize the cascade amplification of antitumor immune response, we report for the first time a TME-responsive nanoplatform through coating oxygen-vacancy-doped MnO_2-x_ onto Cu-doped Fe-based MOF (FCM) for the fabrication of Fe-Cu-MOF@MnO_2-x_ (FCMM) heterojunctions. The introduction of Cu ions in Fe-MOF and the encapsulation of MnO_2-x_ enable FCMM as a high-efficiency sonozyme, achieving enhanced ROS production through heterojunction-mediated sonodynamic activity amplification and Fe/Cu/Mn-ion-triggered multienzyme-mimic activities including Fenton/Fenton-like reaction, GSH depletion, and hypoxia alleviation.

**Results:**

The reversal of the immunosuppressive tumor microenvironment occurs through the ROS-triggered ICD and the enhancement of DC cell maturation. More importantly, the activation of the Mn ions-mediated cGAS-STING pathway further boosts the maturation of DCs. In addition, the released Cu ions can induce cuproptosis, achieving triple amplification of antitumor immune response.

**Conclusion:**

The combination therapy of CDT, SDT, cuproptosis, and cGAS-STING activation via FCMM, achieved complete elimination of primary tumor and significant controlled the growth of distant tumor. This work combines sonocatalytic nanomedicine with immune modulation strategy through integrating ROS amplification, cGAS-STING activation, and cuproptosis effect into a single nanoplatform, providing new insights for the clinical application of sono-immunotherapy.

## Introduction

Cancer immunotherapy provides new insights into clinical tumor therapy, which exploits the host immune system to combat tumor cells 1-5. While there has been notable progress in immune checkpoint blockade (ICB), CAR T-cell therapy, and cancer vaccines, their clinical efficacy remains limited for the majority of patients 6-9. For example, ICB targeting the programmed death-1 (PD-1)/programmed death ligand-1 (PD-L1) axis exhibits unsatisfactory response rates in many solid tumors due to low PD-L1 expression and insufficient tumor-infiltrating lymphocytes 10-12. Similarly, CAR-T and vaccine-based approaches suffer from antigen heterogeneity, immune exhaustion, and poor infiltration within the immunosuppressive tumor microenvironment (TME) 6, 13-15. These barriers result in so-called “immunologically cold” tumors that fail to evoke effective immune responses 16, 17. Consequently, strategies capable of reprogramming immunosuppressive TME and enhancing the immunogenicity of tumor cells are highly desired. Immunogenic cell death (ICD), a special form of cell death accompanied by the exposure of calreticulin (CRT), release of adenosine triphosphate (ATP) and high-mobility group box 1 (HMGB1), has emerged as an effective approach to initiate antitumor immunity 18-20. However, the induction of ICD effect is highly dependent on the level and persistence of intracellular oxidative stress, which is typically low in TME because of limited reactive oxygen species (ROS) generation and abundant antioxidant defense 21-24. Hence, increasing ROS production to induce strong ICD effect is essential for boosting tumor immunotherapy.

Photodynamic, sonodynamic, and chemodynamic therapy (PDT/SDT/CDT) has gained widely attention for inducing tumor cell death and subsequent ICD through amplifying intracellular oxidative stress 25-30. Despite PDT possesses the precise spatial control features, the therapeutic depth of PDT is significantly hindered by the poor tissue penetration of NIR light and the requirement for oxygen, often resulting in unsatisfactory efficacy in hypoxic tumors 29, 31, 32. For comparison, SDT employs ultrasound (US) to activate sonosensitizers, enabling deeper tissue penetration for ROS production 33-37. However, the application of SDT remains limited due to two intrinsic limitations. First, the ROS production efficiency of conventional sonosensitizers is relatively low 38-43, for example, inorganic sonosensitizers usually possess wide bandgaps and suffer from rapid electron-hole recombination, while organic sonosensitizers often show potential phototoxicity 27, 35, 44. Second, TME often exhibits severe biochemical restrictions, including overexpressed GSH for the sacrifice of ROS and hypoxia for the limitation of SDT efficiency, thereby diminishing therapeutic outcomes 45-47. Nanozymes have recently emerged as a promising method to overcome these obstacles by simultaneously augmenting ROS generation and remodeling the TME 48, 49. On the one hand, nanozymes with Fenton/Fenton-like reaction activity can catalyze endogenous H_2_O_2_ into high biotoxicity •OH, realizing synergetic CDT and SDT 50-53. On the other hand, nanozymes possessing GSH-px-like or CAT-like activities can effectively modulate redox homeostasis by depleting intracellular GSH and decomposing H_2_O_2_, thereby alleviating the immunosuppressive and antioxidant characteristics of the TME 54-58. Hence, rationally designing multifunctional sonozymes that integrate efficient sonocatalytic and Fenton-like reactivity, together with TME-responsive behavior, holds great potential for amplifying ROS-induced ICD and improving the immunotherapeutic efficacy of SDT/CDT.

In addition to inducting ICD, activating innate immune pathways also plays a vital role in eliciting durable antitumor immunity. Among them, the cyclic GMP-AMP synthase-stimulator of interferon genes (cGAS-STING) signaling pathway has recently gained considerable attention for its ability to detect dsDNA and IFN-β production 59, 60. Activation of the cGAS-STING pathway boosts DC maturation, improves antigen presentation, and attracts cytotoxic T lymphocytes 61-63. Nevertheless, existing approaches to activate STING depend on external agonists like cyclic dinucleotides, which limited by inadequate cytosolic delivery, quick degradation, and systemic inflammatory reactions 59, 60, 64. Therefore, it is important to develop more controllable and biocompatible approaches to activate the cGAS-STING pathway within tumors. Recent research have revealed that Mn-based nanomaterials provide a promising alternative for modulating innate immunity 59. The Mn^2+^ ions released from Mn-based nanomaterials can bind to cGAS, enhance its enzymatic activity, and trigger downstream STING signaling, thereby amplifying IFN-β secretion and promoting robust antitumor immune responses 61, 65, 66. Moreover, Mn-based nanomaterials are capable of depleting GSH and alleviating hypoxia through GSH-px-like and CAT-like activities, thus remodeling the immunosuppressive TME 67, 68. Therefore, integrating the sonosensitizer and nanozyme activities into Mn-based nanomaterials enables the simultaneous induction of ROS-induced ICD and Mn^2+^-triggered cGAS-STING pathway activation. Such a dual-functional strategy couples catalytic ROS amplification with innate immune stimulation, achieving cascade immune amplification for effective cancer immunotherapy.

**Scheme 1:**
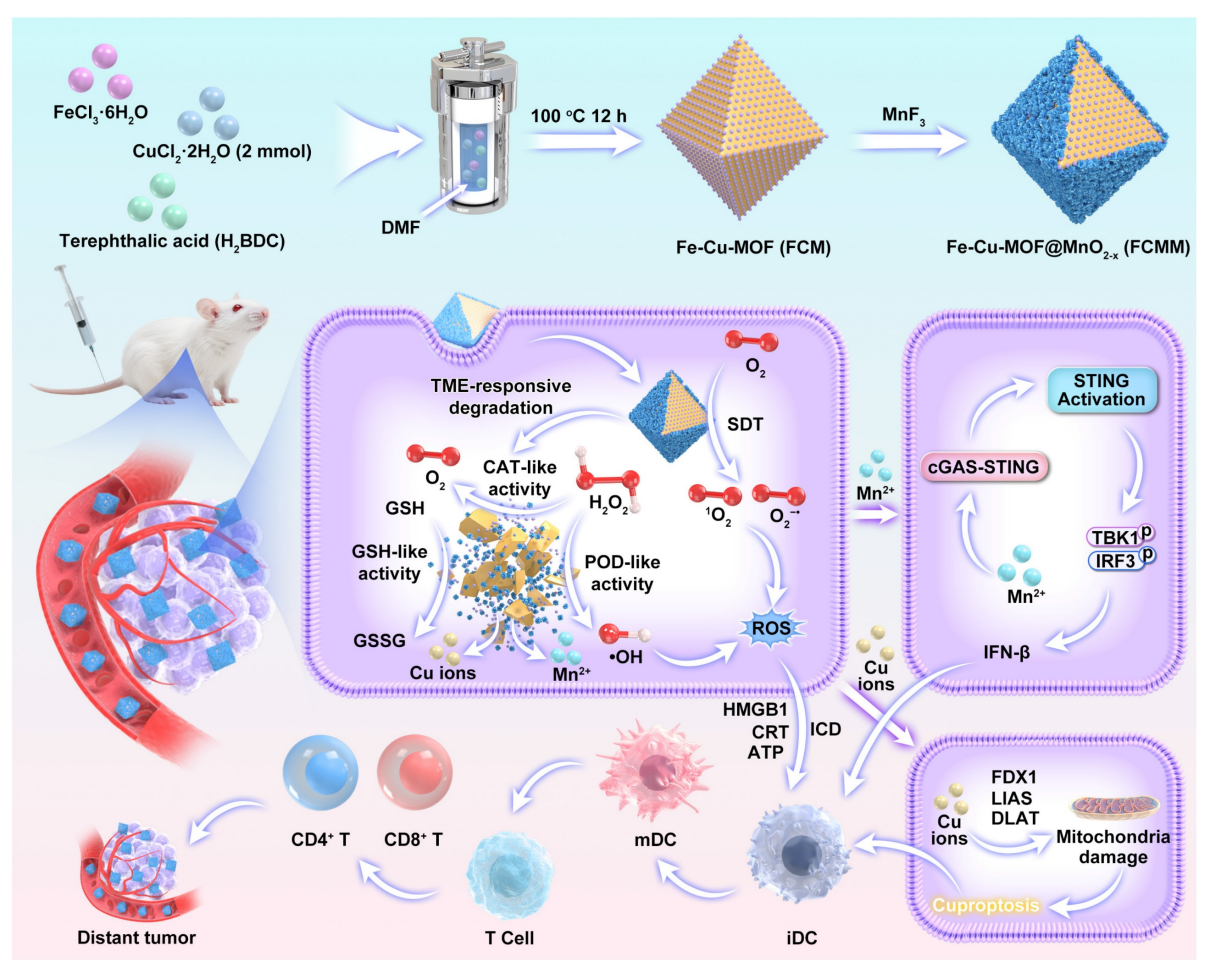
A schematic diagram of the fabrication of FCMM for heterojunction-amplified sono-immunotherapy through activating tumor-specific cuproptosis and cGAS-STING pathway. The MnO_2-x_ coating forms a heterojunction with Fe-Cu-MOF, promoting charge separation and enhancing catalytic efficiency under US irradiation. FCMM exhibits TME-responsive degradation behavior, which can release Mn^2+^ to activate cGAS-STING pathway and Cu^+^/Cu^2+^ to trigger cuproptosis. ROS-mediated ICD, Cu-induced cuproptosis, and Mn-triggered cGAS-STING activation based on FCMM nanoplatforms lead to complete ablation of primary tumors and suppression of distant tumor growth.

In this work, we reported for the first time the rational design of a multifunctional nanoplatform for cascade amplification of antitumor immune response through integrating Cu-doped Fe-based metal-organic frameworks (FCM) with oxygen vacancy-doped MnO_2-x_ sonozymes (Scheme 1). The incorporation of Cu^2+^ into the Fe-MOF not only modulates the electronic structure and facilitates redox cycling between Fe^2+^/Fe^3+^ and Cu^+^/Cu^2+^ pairs, thereby accelerating electron transfer and enhancing both sonodynamic and Fenton-like catalytic reactions, but also introduces a Cu-dependent cytotoxic pathway (cuproptosis), characterized by mitochondrial protein aggregation and oxidative stress, which further promotes DC maturation and antigen presentation. Meanwhile, the MnO_2-x_ coating forms a heterojunction with Fe-Cu-MOF (FCMM), promoting charge separation and enhancing catalytic efficiency under US irradiation. More importantly, the FCMM system exhibits TME-responsive degradation behavior, which can release Mn^2+^ to activate cGAS-STING pathway and Cu^+^/Cu^2+^ to trigger cuproptosis after the completion of SDT. Finally, ROS-mediated ICD, Cu-induced cuproptosis, and Mn-triggered cGAS-STING activation based on FCMM nanoplatforms lead to complete ablation of primary tumors and suppression of distant tumor growth. This work provides valuable insights into the development of intelligent nanoplatforms for tumor eradication and long-term immune protection through heterojunction and cGAS-STING activation co-enhanced sono-immunotherapy.

We utilized a rapid and facile synthesis method to prepare Fe-MOF using FeCl_3_•6H_2_O and terephthalic acid (H_2_BDC) as the precursors and DMF as the reaction solvent. The successful preparation of Fe-MOF was demonstrated by TEM image presented in Figure S1, which revealed that the size of Fe-MOF was approximately 200 nm. We also utilized DLS to measure the size of Fe-MOF, which was also about 200 nm (Figure S2). The presence of Fe, C, and O elements in Fe-MOF can be verified by the survey XPS spectrum (Figure S3). Two peaks at 710.2 eV and 725.4 eV corresponded to Fe 2p_3/2_ and Fe 2p_1/2_ can be detected in the high-resolution Fe 2p spectrum, which were deconvoluted into Fe^2+^ and Fe^3+^ species. The C 1s spectrum showed two components including C-C/C=C and C-O bonds. These findings clearly provided evidence for the successful synthesis of Fe-MOF.

## Results and Discussion

**Figure 1:**
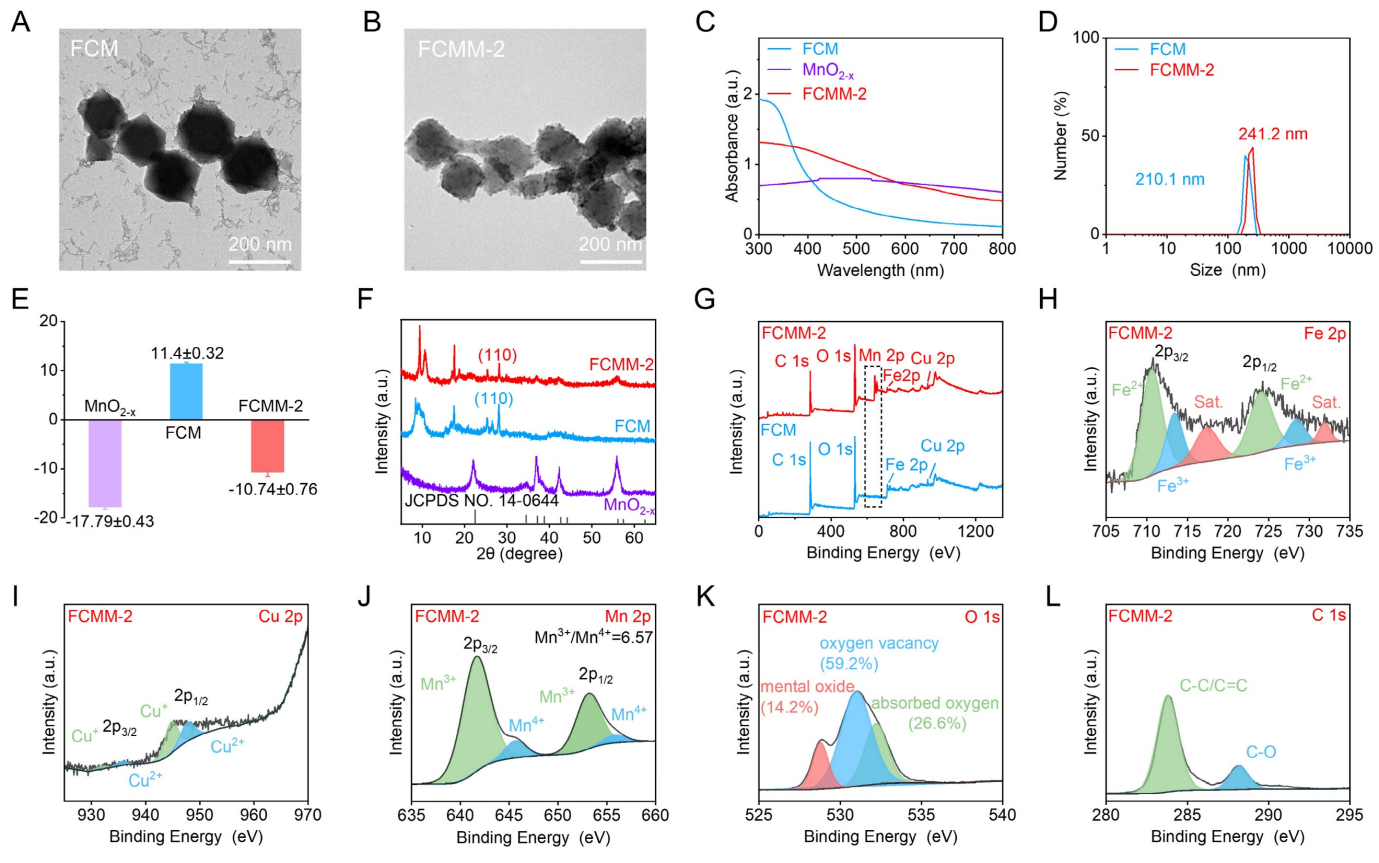
**Preparation and Characterization of FCMM-2.** (A-B) TEM images of FCM and FCMM-2. (C-L) Absorption spectroscopy (C), hydrodynamic diameter (D), Zeta potential measurements (E), XRD patterns (F), Survey XPS (G), high-resolution Fe 2p (H), Cu 2p (I), Mn 2p (J), O 1s (K), C 1s (L) spectra of FCMM-2. Data are presented as the mean ± SD. (n = 3).

TEM images showed that FCM exhibited a well-dispersed octahedral morphology with an average size of approximately 200 nm (Figure 1A), which was consistent with the hydrodynamic diameter determined by DLS measurements (210 nm) (Figure 1D). The hydrodynamic diameter of Fe-MOF was similar to that of FCM, suggesting that Cu doping did not affect the size of Fe-MOF. XRD pattern of Fe-MOF showed characteristic diffraction peaks at 2θ = 9.3°, 10.7°, and 16.8° (Figure S4). In contrast, XRD pattern of FCM showed characteristic peaks at 2θ = 9.1°, 10.4°, 16.6°, and 28.1°. The appearance of new peaks of FCM compared to Fe-MOF confirmed the successful incorporation of Cu ions. Moreover, XPS spectrum of FCM not only contained signals from Fe, C, and O but also displayed Cu elements (Figure 1G). The high-resolution Cu 2p spectrum showed Cu 2p_3/2_ and Cu 2p_1/2_ peaks, which were deconvoluted into Cu^2+^ and Cu^+^ species (Figure S5). Collectively, these results verified the successful introduction of Cu ions and the formation of FCM.

Considering that MnO_2-x_ demonstrated significant potential in cancer treatment by integrating CDT and cGAS-STING pathway activation, we proceeded to fabricate heterojunctions by synthesizing MnO_2-x_- modified FCM. To obtain MnO_2-x_ with good sonodynamic activity, we prepared oxygen-vacancy-doped MnO_2-x_ nanoflowers by utilizing MnF_3_ as the precursor. During the disproportionation reaction of MnF_3_, a large number of oxygen vacancies are generated, which reduces the bandgap of MnO_2-x_ and enhances its sonodynamic activity. The successful formation of MnO_2-x_ was initially confirmed by XPS analysis, which indicated the presence of Mn and O elements (Figure S6). Mn ions were identified in two oxidation states, including Mn^3+^ and Mn^4+^. The crystal structure was further examined by XRD pattern (Figure 1F). The diffraction signal observed in the as-synthesized MnO_2-x_ aligned well with the standard pattern of MnO_2-x_ structure, verifying the successful formation of MnO_2-x_ nanoflowers.

Subsequently, MnO_2-x_ was in-situ assembled on the surface of FCM to construct the Fe-Cu-MOF@MnO_2-x_ heterojunctions (FCMM). To determine the optimal SDT and CDT performances of FCMM, the feeding ratio of FCM to MnO_2-x_ varied from 1:1, 3:1, to 6:1. The three FCMM were denoted as FCMM-1, FCMM-2, and FCMM-3 according to the Cu doping ratio, respectively. TEM image of FCMM-2 revealed a morphology similar to that of FCM (Figure 1B), but with additional particulate matter deposited on the surface, which can be attributed to the successful loading of MnO_2-x_. The average size of FCMM-2 was approximately 200 nm, while the hydrodynamic diameter was measured to be 241.2 nm (Figure 1D). The measured size of FCMM-2 was larger than that of FCM, which could be attributed to the loading of MnO_2-x_. For the FCMM with different mass ratios, the particle sizes remained largely consistent (Figure S7). To verify the successful synthesis of FCMM-2, XRD was employed to analyze the diffraction patterns. XRD pattern of FCMM-2 exhibited a weak diffraction peak at 2θ = 55.8° (Figure 1F), consistent with the characteristic peak of MnO_2-x_ structure. Additionally, a characteristic peak around 25°, attributed to the [110] crystal plane of FCM, was observed, confirming the successful assembly of MnO_2-x_ onto the FCM surface. UV-vis absorption spectrum and Zeta potential measurements provided further evidence. The UV-Vis spectra showed a distinct difference between FCM, MnO_2-x_, and FCMM-2 (Figure 1C). The characteristic absorption peaks of FCMM-2 contained that of FCM and MnO_2-x_, indicating the successful synthesis of FCMM-2. The Zeta potential of MnO_2-x_, FCM, and FCMM-2 were measured as -17.79 ± 0.43 mV, 11.4 ± 0.32 mV, and -10.76 ± 0.76 mV, respectively (Figure 1E), suggesting that the formation of heterojunctions could be the electrostatic attraction between negatively charged MnO_2-x_ and positively charged FCM.

To investigate the chemical composition of FCMM-2, XPS measurements were conducted. XPS survey spectra of FCM and FCMM-2 confirmed the presence of Fe, Cu, O, and C elements (Figure 1G). Notably, Mn was exclusively detected in FCMM-2. A similar elemental profile was observed for FCMM with different mass ratios (Figure S10). The high-resolution Mn 2p spectrum exhibited two main peaks corresponding to Mn 2p_3/2_ and Mn 2p_1/2_ (Figure 1J). The fitted peaks at binding energies of 641.9 eV and 653.4 eV were assigned to Mn^3+^, while those at 643.9 eV and 654.5 eV were attributed to Mn^4+^. By comparing the three types of FCMM prepared with different mass ratios of FCM to MnF_3_, it was found that the proportion of Mn^3+^ in FCMM-2 was higher than that in the other two FCMM (Figure S11A, B). This might give FCMM-2 better POD-like activity. Similarly, the Fe 2p spectrum showed characteristic doublets for Fe 2p_3/2_ and Fe 2p_1/2_ (Figure 1H). The peaks located at 710.2 eV and 723.2 eV were deconvoluted into contributions from Fe^2+^ and Fe^3+^ species. The high-resolution Cu 2p spectrum could be deconvoluted into contributions from Cu^+^ and Cu^2+^ species (Figure 1I). The O 1s spectrum could be fitted with three components, which were assigned to lattice metal oxide, oxygen vacancies, and surface-adsorbed oxygen species (Figure 1K). The presence of oxygen vacancies in MnO_2-x_ could facilitate electron transition and thereby enhanced the efficacy of SDT. More importantly, compared with the other two types of FCMM, FCMM-2 has a higher content of oxygen vacancies (Figure S11C, D), which will enable it to have better SDT activity than FCMM-1 and FCMM-2. Meanwhile, the C 1s spectrum was deconvoluted into two peaks attributed to C-C/C=C and C-O bonds (Figure 1L). As shown in Figure S12, no significant precipitation was observed in the FCMM-2 solution over a period of 7 days, indicating good stability of FCMM-2. Collectively, these findings provided further evidence for the successful synthesis of FCMM-2.

**Figure 2:**
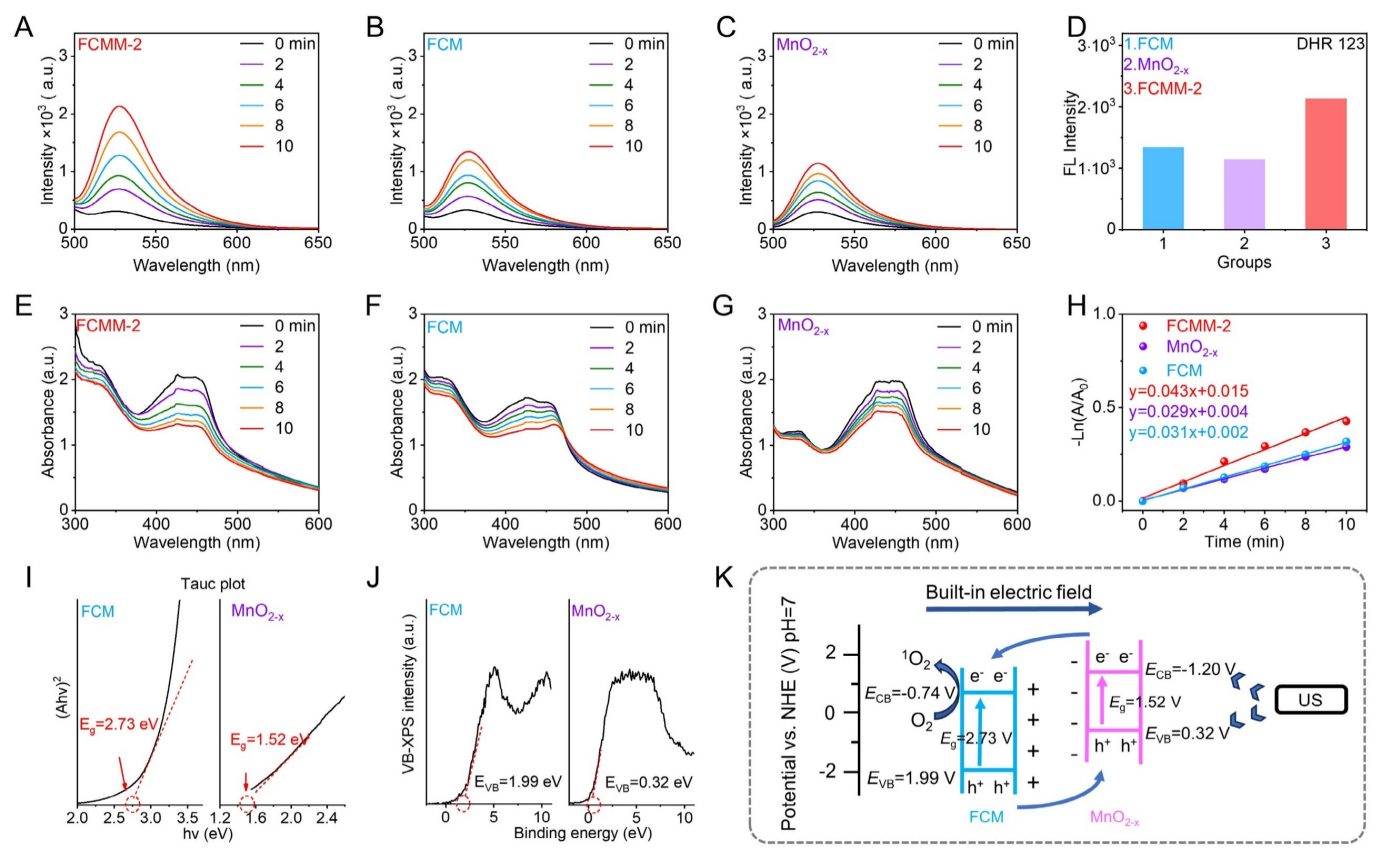
** Amplified Sonodynamic Performance of FCMM-2.** (A-D) The generation efficiency of O_2_^-•^ was evaluated using fluorescence spectroscopy for FCMM-2, FCM and MnO_2-x_. (E-H) ROS generation efficiency was assessed through absorption spectroscopy for FCMM-2, FCM and MnO_2-x_. (I, J) Bandgap and valence band characterization of FCM and MnO_2-x_ from Tauc plot and XPS-VB measurements. (K) Schematic diagram of the built-in electric field of the FCMM-2 heterojunctions to show the electron migration and related energy levels.

The ability of FCMM-2 to generate O_2_^-•^ under US irradiation was first investigated using DHR 123 as a fluorescent probe. Fluorescence spectra indicated that O_2_^-•^ was generated under US irradiation in all three systems: FCMM-2 (Figure 2A), FCM (Figure 2B), and MnO_2-x_ (Figure 2C). Among them, the FCMM-2 system exhibited the strongest fluorescence intensity (Figure 2D), suggesting the highest O_2_^-•^ production efficiency. Subsequently, the ROS generation capability of FCMM-2 under US was evaluated using DPBF as a probe. The changes in the absorption spectra of FCMM-2, FCM, and MnO_2-x_ under US irradiation for different durations (0-10 min) are shown in Figure 2E-G, respectively. We observed a reduction in the characteristic absorption signal of DPBF, indicating a certain ROS generation capability in all three FCMM. The characteristic peak of DPBF decreased most significantly for FCMM-2, indicating that heterojunction formation strengthens the effect of SDT. By calculating the ROS generation rates, it was found that after 10 min of US, the reaction rate of FCMM-2 was 1.42 and 1.73 times higher than that of FCM and MnO_2-x_, respectively. (Figure 2H). Collectively, the results demonstrated that coating MnO_2-x_ can effectively enhance the sonodynamic performance of single-component sonosensitizers. To explore the optimal performance of FCMM heterojunctions with different mass ratios, the US-activated ROS generation ability of these kinds of FCMM was evaluated and compared. Fluorescence spectra revealed that both FCMM-1 and FCMM-3 exhibited the ability to generate O_2_^-•^ (Figure S13). Among them, FCMM-2 exhibited the fastest O_2_^-•^ generation rate. In addition to O_2_^-•^, FCMM-2 also demonstrated the most prominent ROS production efficiency when utilizing DPBF as the ROS probe for the detection of ROS production (Figure S14). The above data indicated that FCMM-2 possessed the highest SDT performance compared with the other FCMM prepared from different mass ratios of FCM to MnO_2-x_. This is related to the charge separation efficiency after the heterojunction construction. We compared and analyzed the charge transfer characteristics of three different FCMM heterojunction samples through electrochemical impedance spectroscopy. As shown in Figure S15, the impedance value of FCMM-2 was significantly lower than that of the other two FCMMs. This indicates that FCMM-2 can more effectively promote the separation of electrons and holes, and ultimately increase the generation of reactive oxygen species.

To elucidate the mechanism underlying the enhanced sonodynamic activity of FCMM-2, the bandgap and valence band positions of FCM and MnO_2-x_ were measured. Based on Tauc plot analysis, the bandgaps of FCM and MnO_2-x_ were calculated as 2.73 eV and 1.52 eV, respectively (Figure 2I). The valence band positions of FCM and MnO_2-x_ were measured via XPS-VB spectroscopy and found to be 1.99 eV and 0.32 eV, respectively (Figure 2J). The CB positions of FCM and MnO_2-x_ were determined to be -0.74 eV and -1.20 eV, respectively, based on experimentally obtained band gap and valence band values. Owing to their opposite zeta potentials, a well-defined electron transfer pathway was established. Within this scheme, electrons from the CB of MnO_2-x_ were directionally transferred to the CB of FCM under the influence of an interfacial built-in electric field. This process effectively facilitated the spatial separation of charge carriers and significantly suppressed the recombination of electron-hole pairs. As a result, the lifetime of the charge carriers was extended, leading to an enhancement in the overall reaction efficiency.

**Figure 3:**
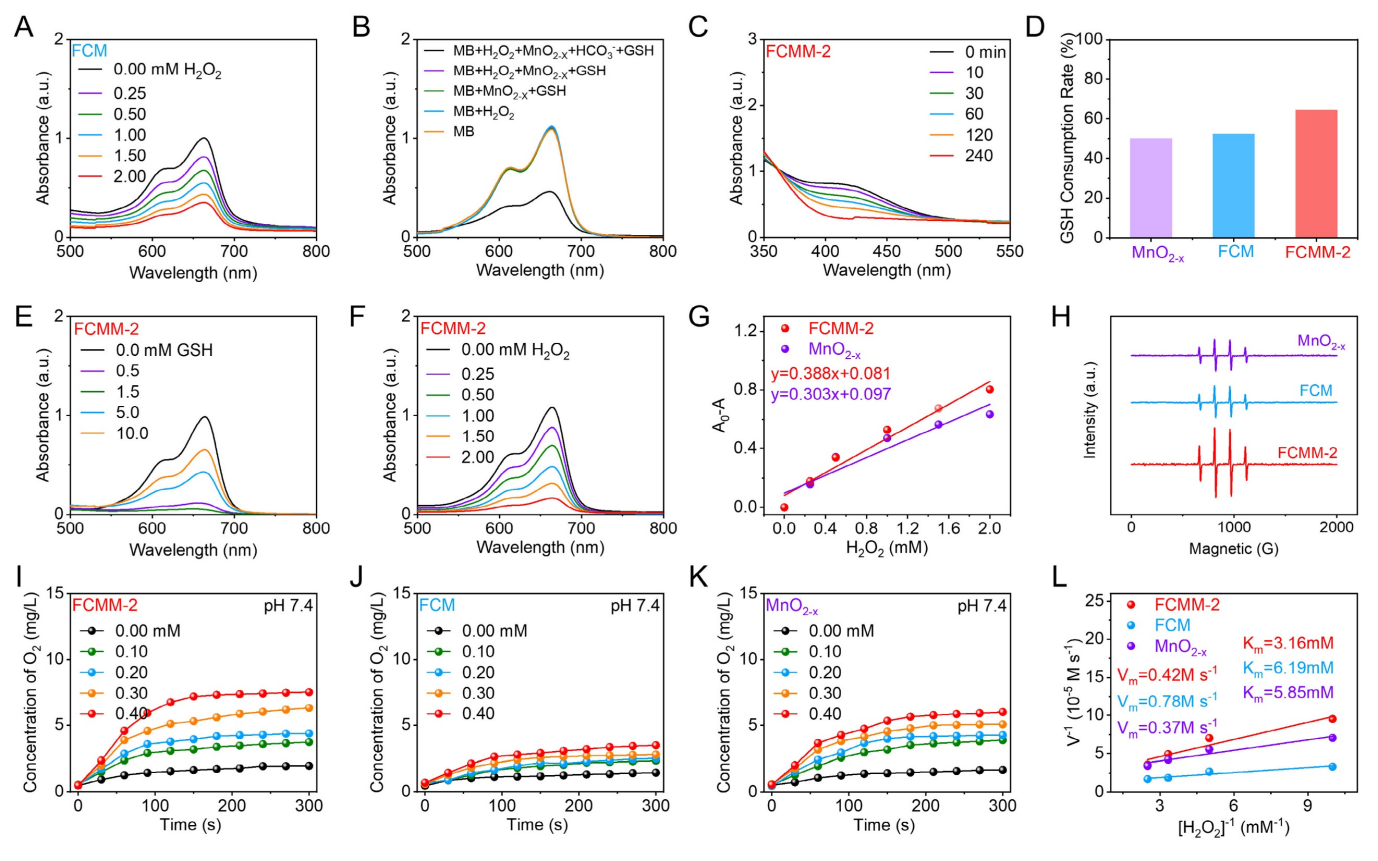
** Heterojunction-Enhanced Enzyme-Mimicking Activities of FCMM-2.** (A) Absorption spectra of the POD-like activity evaluation of FCM. (B) UV-vis absorption spectra of MB after degradation by the MnO_2-x_ mediated Fenton-like reaction in different solutions. (C, D) GSH-px-mimic catalytic activity evaluation of FCMM-2, FCM, and MnO_2-x_. (E) Degradation of MB by FCMM-2 after being treated with different concentrations of GSH. (F) Degradation of MB by FCMM-2 in H_2_O_2_ solutions with different concentrations. (G) Comparative evaluation of POD-mimic catalytic activities between FCMM-2 and MnO_2-x_. (H) ESR spectra of FCMM-2, FCM, and MnO_2-x_. (I-L) Comparative characterization of CAT-mimic catalytic activities among FCMM-2, FCM, and MnO_2-x_ at pH 7.4.

The ability of FCM to generate •OH via a Fenton-like reaction was first investigated by monitoring the degradation of MB. First, when only H_2_O_2_ and MnO_2-x_ were added, we found that the absorption of MB did not show any significant change (Figure 3B). This can rule out the possibility that the decomposition of H_2_O_2_ or the adsorption of substances affected the degradation of MB. As depicted in Figure 3A, a continuous decrease in the characteristic absorption peak of MB was observed with increasing H_2_O_2_ concentration, indicating that FCM exhibited intrinsic Fenton/Fenton-like activity owing to the presence of Cu^+^ and Fe^2+^. However, when MnO_2-x_ was incubated directly with H_2_O_2_ in NaHCO_3_/CO_2_ buffer, no significant change in the characteristic MB absorption peak was detected, indicating that MnO_2-x_ alone could not directly produce •OH through a Fenton-like reaction (Figure 3B). When MnO_2-x_ was pre-incubated with GSH for 15 minutes before the same treatment, the characteristic absorption peaks of MB showed a significant weakening. These phenomena indicated that the POD-like activity of MnO_2-x_ could only be activated upon the addition of GSH, which converts Mn^4+^ and Mn^3+^ to Mn^2+^ and thereby initiated a Fenton-like reaction.

A DTNB-based colorimetric method was employed to assess the GSH consumption capability of MnO_2-x_. As shown in Figure S16, MnO_2-x_ induced a reduction in the characteristic absorption peak of DTNB, indicating its ability to deplete GSH. Compared to MnO_2-x_ alone, the FCMM-2 composite exhibited a more pronounced decrease in the GSH absorption peak within the same time period, along with a higher GSH consumption rate (Figure 3C, D), indicating its enhanced GSH-Px-mimic catalytic activity. Subsequently, the POD-like activity enhanced by GSH consumption was examined using FCMM-2. As depicted in Figure 3E, a reduction in the characteristic peak of the MB indicator was detected after the addition of GSH-treated FCMM-2, which was attributed to the Fenton-like reaction mediated by Mn^2+^ released from MnO_2-x_ upon GSH stimulation. Furthermore, the decrease in the MB absorption peak became more significant with increasing GSH concentration. However, when the GSH concentration exceeded 1.5 mM, the reduction of the MB peak was markedly suppressed, suggesting that excess GSH scavenges the generated •OH, thereby limiting the POD-like activity. Next, an MB degradation assay was conducted in a NaHCO_3_/CO_2_ buffer system containing H_2_O_2_, with the addition of 1.5 mM GSH-pretreated FCMM-1, FCMM-2 and FCMM-3, respectively (Figure 3F and S17). The FCMM-2 system exhibited a faster MB degradation rate (Figure 3G), indicating a higher •OH generation efficiency. These results demonstrated that FCMM-2 not only possessed the intrinsic Fenton/Fenton-like activity of the FCM core but also acquired a GSH-responsive •OH generation capability from the MnO_2-x_ shell, resulting in a synergistic enhancement of POD activity. To further verify •OH generation, ESR measurements were conducted. As depicted in Figure 3H, FCMM-2 effectively produced •OH, and its ESR signal intensity was significantly enhanced compared to those of FCM and MnO_2-x_. Moreover, among the composites with different ratios, FCMM-2 exhibited a superior reaction rate compared to FCMM-1 and FCMM-3, indicating the highest POD-like activity. Therefore, FCMM-2 was selected for subsequent experimental studies.

In addition to exhibiting POD-mimic and GSH-px-mimic activities, the CAT-mimic activities of FCMM-2, FCM and MnO_2-x_ were further compared. The O_2_ generation efficiency of these samples was systematically evaluated under varying H_2_O_2_ concentrations (0-0.4 mM) and different pH conditions (Figure 3I-K). At pH 7.4, all three samples produced increasing amounts of O_2_ with elevating H_2_O_2_ concentrations, demonstrating a distinct H_2_O_2_-dependent behavior. As shown in Figure 3L, the calculated maximum reaction rate and Michaelis constant collectively indicated that FCMM-2 possessed higher CAT-mimic activity compared to FCM and MnO_2-x_. Besides the concentration-dependent behavior, FCMM-2 also showed pH-dependent CAT-mimic catalysis (Figure S18), exhibiting higher O_2_ generation efficiency at pH 7.4 than at pH 6.0 and 6.5. Similar pH-dependent trends were observed for FCM and MnO_2-x_ (Figure S19, S20). Collectively, these results indicated that the incorporation of MnO_2-x_ significantly enhanced CAT-mimic activity, enabling more efficient O_2_ production and augmenting the multi-enzyme mimicking activities, thereby laying a foundation for further research in antitumor applications.

**Figure 4:**
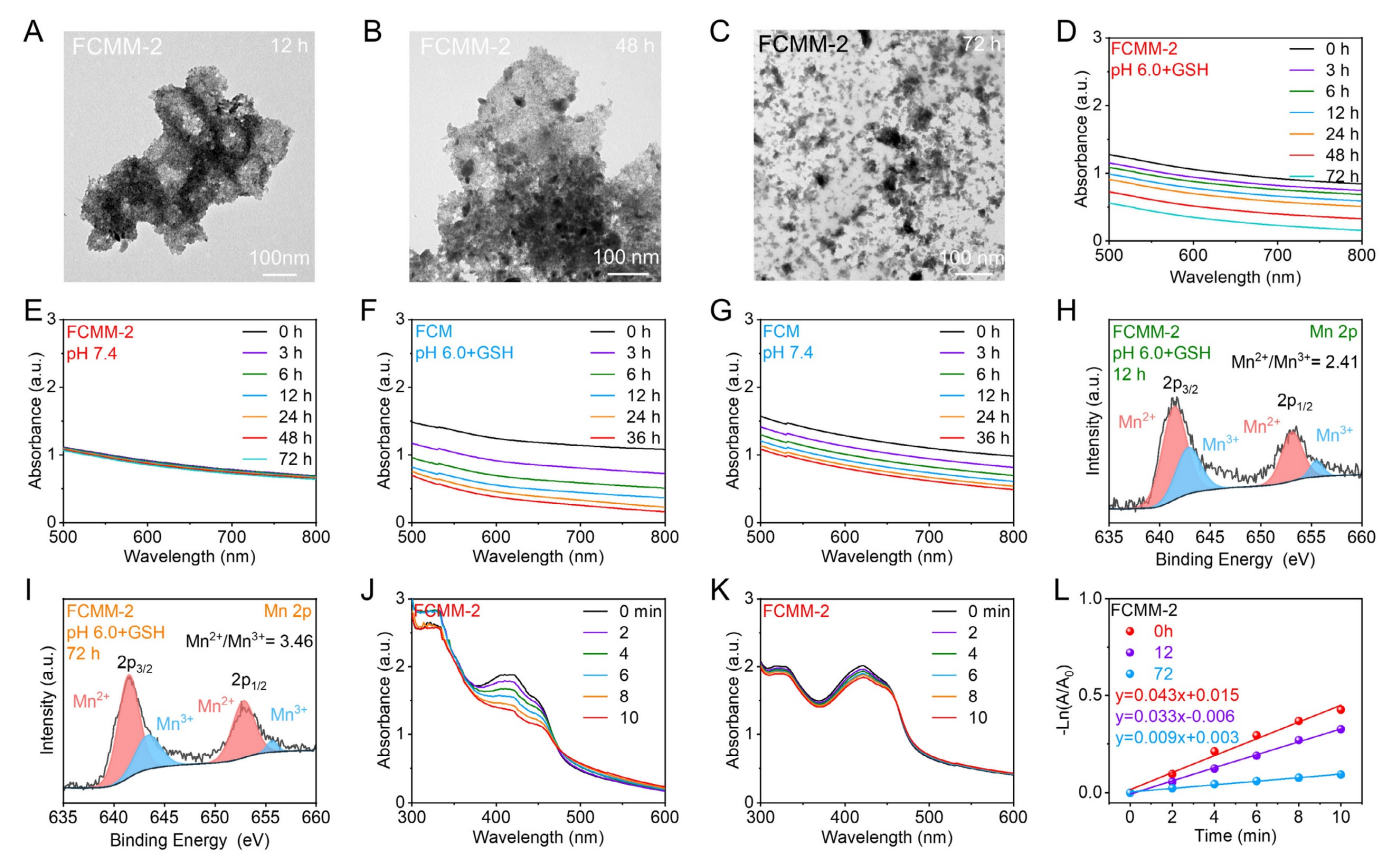
** TME-Triggered Degradation Behavior of FCMM-2.** (A-C) TEM images of FCMM-2 incubated at pH 6.0 for various times. (D, E) Absorption spectra of FCMM-2 at different time in varied solution (pH 6.0 + GSH or pH 7.4). (F, G) Absorption spectra of FCM at different time in varied solutions (pH 6.0 +GSH or pH 7.4). (H, I) The high-resolution Mn 2p spectra of FCMM-2 after different periods of degradation. (J-L) The sonodynamic activity of FCMM-2 after different periods of degradation.

The degradability of nanomaterials is a crucial indicator in assessing their biological applications. TME is characterized by a high level of H_2_O_2_ and GSH, as well as mild acidity, providing a conducive environment for the responsive degradation of nanomaterials. Consequently, we assessed the TME-responsive degradation potential of FCMM-2. Firstly, we investigated the degradation of FCMM in a simulated neutral environment (pH 7.4, PBS solution). Figure 4E showed the absorption spectra of FCMM-2 after different incubation times, indicating that FCMM has good stability. As a control, we also investigated the degradation of FCM and found that the absorbance of FCM gradually decreased with the extension of incubation time, suggesting that FCM may have degraded (Figure 4G). These datas demonstrated that MnO_2-x_ loading could effectively improve the stability of FCM and avoid the inevitable release of Cu ions in normal tissues. The TME-responsive degradation behavior of FCMM-2 was then investigated, which was strongly dependent on pH and GSH levels. As shown in Figure 4D, F, the absorbance of FCM and FCMM-2 were significantly weakened with the increase of time, which indicated that they could gradually degrade in pH 6.0 solution containing 5 mM GSH. To further explain the TME-responsive degradation behavior of FCMM, the microscopic morphology and structure of FCMM-2 at different times (12, 48, 72 h) were analyzed by TEM and XRD. As depicted in Figure 4A-C, after 12 hours of storage, the octahedral structure of FCMM-2 partially collapsed and agglomeration of FCMM-2 occurred. A number of FCMM-2 fragments appeared at 48 h and more fragments were formed at 72 h. The TEM images indicated that FCMM-2 has excellent TME response degradation performance. Subsequently, we performed XRD measurement on FCMM-2 stored for 12 h and 72 h, respectively (Figure S21). As expected, the intensity of the diffraction peak of FCMM-2 weakened as the incubation time increased, indicating that FCMM-2 degraded progressively over a three-day period. We then examined the valence alterations of Mn ions as FCMM-2 degraded by conducting high-resolution Mn 2p X-ray photoelectron spectra measurements. As presented in Figure 4H, I, the ratio of Mn^2+^/Mn^3+^ increased after 12 and 72 hours of incubation in the presence of GSH, confirming the degradation of FCMM-2 and the reduction of Mn^4+^ to Mn^2+^/Mn^3+^. Upon verifying the TME-responsive degradation properties of FCMM-2, we proceeded to examine its changes in sonodynamic activity. FCMM-2 maintained its sonodynamic therapy (SDT) activity within the initial 12 hours of degradation (Figure 4J), but it was completely lost after 72 hours (Figure 4K). As shown in Figure 4L, the ^1^O_2_ generation efficiency was markedly reduced from the initial 0.043 to 0.033 and 0.009 min^-1^, respectively. These results show that FCMM-2 demonstrates good stability in the early stages and can complete sonodynamic therapy. Over time, its gradual degradation assists in the elimination of the material from the body. Collectively, these results demonstrated the TME-dependent degradation behavior of FCMM-2. This intelligent responsive nanoplatform ensures effective tumor therapy without causing harm to normal tissues, indicating its promising biosafety profile.

**Figure 5:**
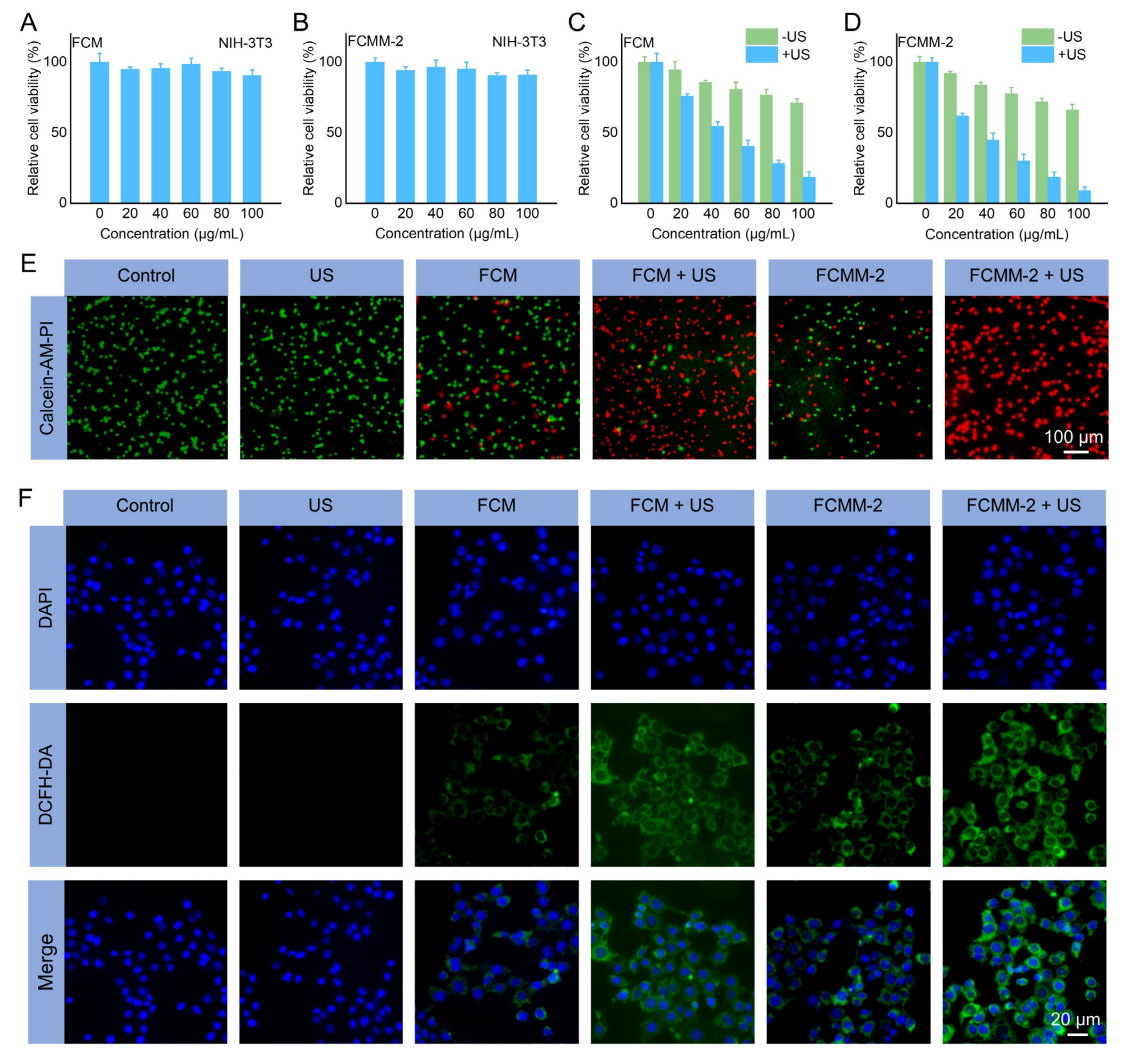
**Antitumor Effects of FCMM-2 at the Cellular Level.** (A-D) Cytotoxicity of FCM or FCMM-2 against NIH-3T3 or 4T1 cells with or without US treatment. (E, F) Live/dead and ROS staining of 4T1 cells after different treatment. Data are presented as the mean ± SD. (n = 6).

Encouraged by the excellent performance of FCMM-2, its antitumor effects at the cellular level were further investigated. Cellular uptake experiments demonstrated that the FCMM-2 can be internalized by tumor cells (Figure S22). The cytotoxicity of FCM and FCMM-2 toward NIH-3T3 normal cells and breast cancer cells (4T1) was assessed through the MTT assay. As shown in Figure 5A, B, neither FCM nor FCMM-2 exhibited significant cytotoxicity against NIH-3T3 cells even after 24 h of incubation at a concentration of 100 μg/mL, indicating their good biocompatibility and minimal harm to normal tissues. In contrast, at the same concentration (100 μg/mL), the survival rates of 4T1 tumor cells treated with FCM and FCMM-2 were approximately 70% (Figure 5C, D), suggesting a certain tumor-killing effect, which can be attributed to the POD like enzyme activities triggered by FCM and FCMM-2. Following the addition of US treatment (50 kHz, 1.0 W/cm^2^, 10 min), the survival rate of 4T1 cells treated with FCM and FCMM-2 significantly decreased. The cell death rate in the FCMM-2 group was notably higher than that of FCM, confirming that FCMM-2 mediated CDT enhances SDT therapy has an excellent antitumor effect. To further elucidate the anti-tumor effect of FCMM-2, we performed a cell apoptosis assay (Figure S23). Compared to the control group (1.62%), the late apoptosis cell ratio in FCMM-2+US group was 81.76%, indicating that FCMM-2 has a stronger killing ability on tumor cells under US irradiation.

Apart from MTT assay, the therapeutic effect of FCMM-2-mediated CDT enhances SDT therapy was assessed by the Calcein-AM and PI co-staining assay. As depicted in Figure 5E and S24A, the control and US alone group only showed the characteristic green fluorescence of living cells. On the contrary, red fluorescence signals were observed in the FCM, FCMM-2, FCM+US, and FCMM-2+US group. Among them, the intensity of the red fluorescence signal was significantly higher in the FCM+US or FCMM+US group than in the groups treated with FCM or FCMM alone. Notably, the absence of a green signal in the FCMM-2 + US group indicates the successful killing of tumor cells, showing the excellent anti-tumor effect of FCMM-2 with US irradiation. The DCFH-DA staining method was used to observe the production of ROS in 4T1 cells in different treatments groups, aiming to understand the anti-tumor mechanism of FCMM-2 mediated CDT enhances SDT. In Figure 5F and S24B, the cells of the control and US alone groups exhibited no green fluorescence signal, indicating that without the presence of sonosensitizer/nanozyme, ROS production in the cells is minimal. However, in the FCM, FCM+US, FCMM-2, and FCMM-2+US groups, the intensity of green fluorescence signal gradually increased, showing that these treatments could induce ROS production in 4T1 cells. Among them, the green fluorescence signal was the strongest in the FCMM-2 + US group, demonstrating that CDT enhanced SDT therapy mediated by FCMM-2 could effectively induce tumor cell apoptosis through the production of ROS. Furthermore, changes in the mitochondrial membrane potential of tumor cells after various treatments were measured to assess mitochondrial damage. As depicted in Figure S25, the strongest green fluorescence was detected in the FCMM-2 + US group, demonstrating the most severe disruption of mitochondrial function caused by the combined treatment. Collectively, these results indicated that the FCMM-2 heterojunctions can achieve satisfactory antitumor efficacy through multiple synergistic mechanisms.

**Figure 6:**
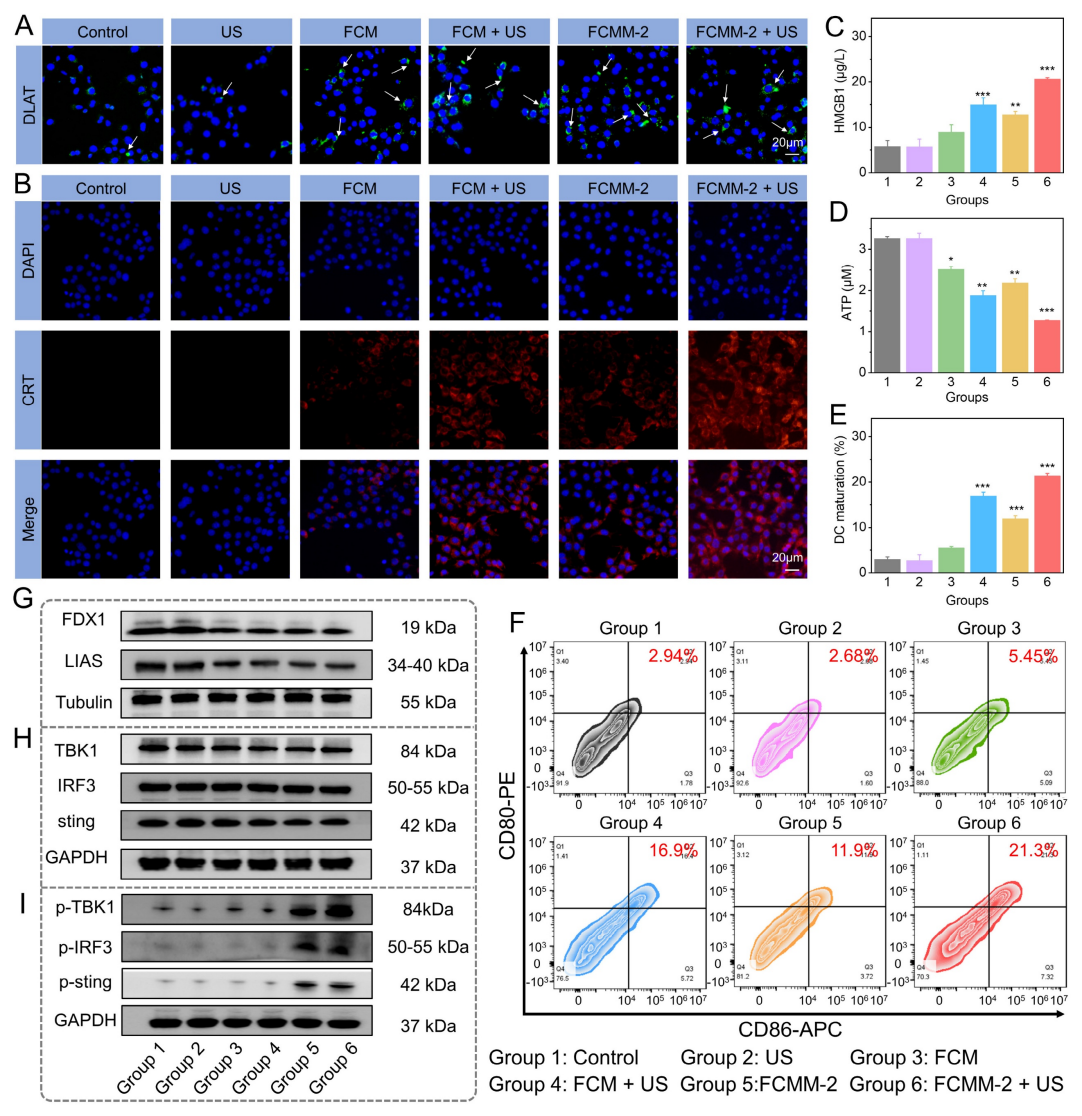
***In Vitro* Anticancer Mechanism of FCMM-2.** (A) DLAT fluorescence images of 4T1 cells after different treatments. (B-D) Levels of CRT, HMGB1, and ATP in 4T1 cells after different treatments. (E, F) Expression of CD80 and CD86 in DCs after different treatments determined by flow cytometry. (G-I) Protein expression of cuproptosis-related and cGAS-STING-related pathway proteins in 4T1 cells after different treatments. Data are presented as the mean ± SD. (n = 3). *p < 0.05, **p < 0.01, and ***p < 0.001.

Following the demonstration of effective *in vitro* therapeutic effectiveness via FCMM-2 induced ROS generation, the associated antitumor mechanisms were further investigated. It is widely recognized that ROS can induce ICD and trigger a robust immune response in tumor cells, leading to the exposure of DAMPs such as CRT, HMGB1, and ATP 24, 34, 69. Accordingly, the ATP, CRT, HMGB1 levels in 4T1 cells after being treated with FCMM-2 were evaluated. First, we employed immunofluorescence staining to observe the expression of CRT on the cell membrane surface of 4T1 cells in different treatment groups (Figure 6B). Compared with the control group, the FCMM-2+US group demonstrated a great red fluorescence signal, indicating that FCMM-2-mediated tumor therapy can cause CRT exposure. Furthermore, a significant decrease in intracellular ATP was observed in the FCMM-2 + US group compared to the control group (Figure 6D), indicating the secretion of ATP into the extracellular region upon combined FCMM-2 and SDT treatment. Similarly, the high levels of HMGB1 were detected in 4T1 cells treated with FCMM-2 under US irradiation, as shown in Figure 6C, suggesting the strongest activation of ICD effect.

Given the existence of Cu ions and the thermo-responsive characteristics of FCMM-2, we investigated the potential for cuproptosis mediated by FCMM-2. The effect of FCMM-2 on cuproptosis was investigated using 4T1 cells. We performed a Western blot analysis to examine the expression levels of cuproptosis-related proteins. As depicted in Figure 6A, a distinct DLAT aggregation was detected in 4T1 cells after treatment with FCM and FCMM-2, indicating that both FCM and FCMM-2 could release Cu ions and successfully induce cuproptosis. A similar result can be also detected in the FCM + US group and the FCMM-2 + US group, demonstrating that the addition of US treatment would not affect the cuproptosis effect induced by FCM and FCMM-2. Then, other representative proteins associated with cuproptosis were investigated. Figure 6G and S26 showed that the expression of LIAS and FDX1 was reduced in 4T1 cells after incubation of FCMM-2, suggesting that cuproptosis mediated by FCMM-2 led to a decrease in iron-sulfur cluster proteins.

Besides Cuproptosis, Mn^2+^ is known to influence cancer cells by activating the STING pathway 59, 65, 70. The expression of cGAS-STING pathway-related proteins of different treatment group was examined. The expression signals of TBK1, IRF3, and STING were similar in the FCMM-2 and FCMM-2 + US groups compared to the control group (Figure 6H and S27), indicating that t the lack of regulatory effects of these treatments on the expression of these proteins. In contrast, the levels of p-IRF3, p-TBK1, and p-STING significantly increased in the FCMM-2 + US group compared to the control group (Figure 6I). These results demonstrated that the released Mn^2+^ stimulated cGAS, enhancing cGAMP production and subsequently activating the STING signaling pathway.

It is established that activation of both the ICD, cuproptosis effect, and cGAS-STING pathway can promote DC maturation and subsequent activation of cytotoxicity T lymphocytes 16, 71. On this basis, following different treatment of 4T1 cells, supernatants were collected for stimulating dendritic cell cultures. The maturation status of DCs was then assessed by measuring the quantification of CD86 and CD80 expression levels. Among all experimental groups, the FCMM-2 + US group exhibited the highest proportion of CD80^+^CD86^+^ DCs (Figure 6E, F), demonstrating that FCMM-2 can induce a potent immune response through the integration of SDT, CDT, cuproptosis, and cGAS-STING pathway activation.

**Figure 7:**
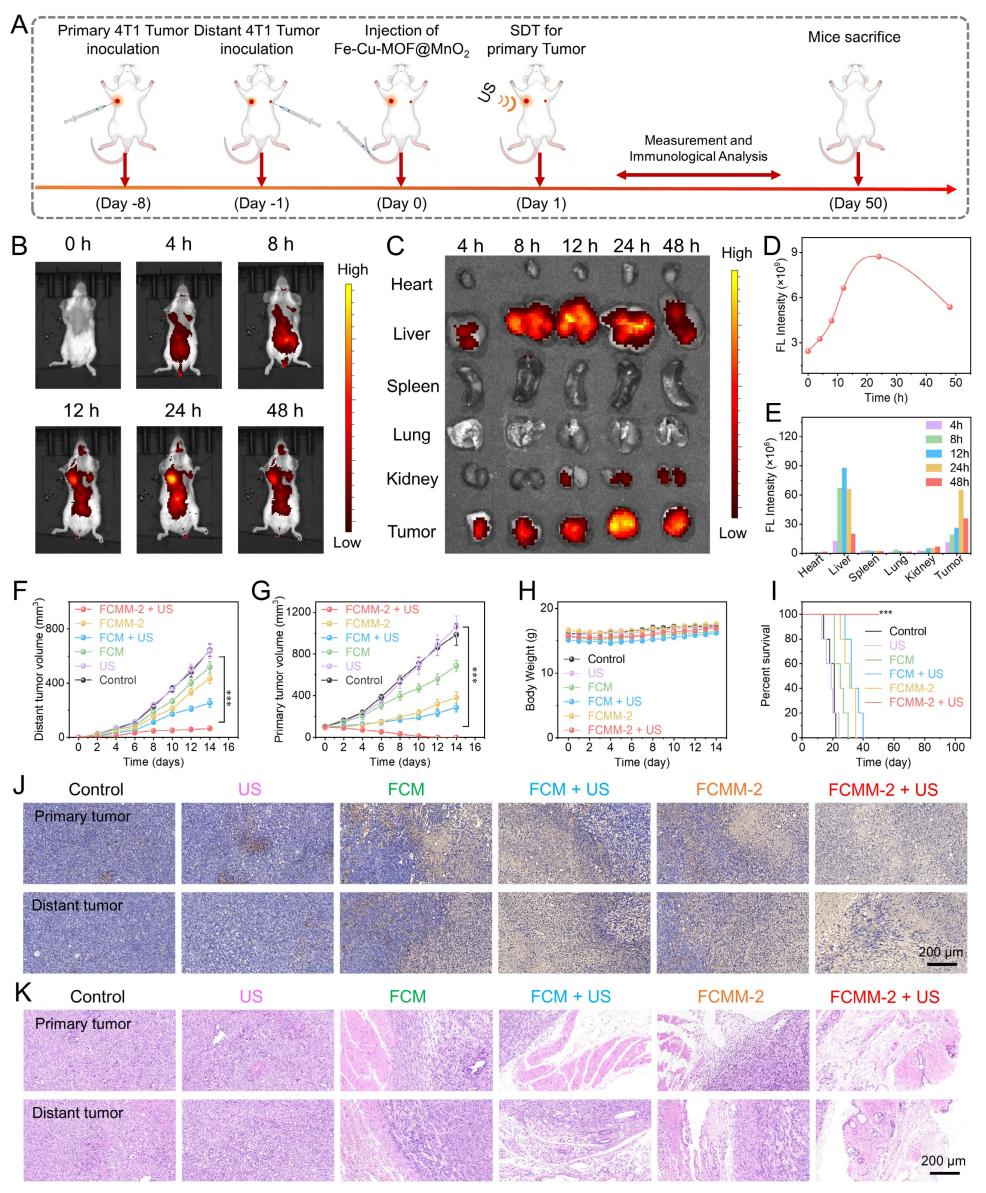
***In Vivo* Antitumor Efficacy of FCMM-2.** (A) Schematic diagram of the *in vitro* anticancer therapy of FCMM-2. (B-E) NIR fluorescence imaging of mice at different times after intravenous injection of FCMM-2. (F-I) Volumes of primary and distant tumors, body weight, and survival rate in mice after different treatments. (J, K) H&E and TUNEL staining of tumors in mice after different treatments. Data are presented as the mean ± SD. (n = 5). ***p < 0.001.

Encouraged by the outstanding therapeutic effects of FCMM-2-mediated combination therapy, which were demonstrated by the *in vitro* experiments, we subsequently evaluated the antitumor efficacy of FCMM-2 *in vivo*. We established a bilateral tumor model in mice, and FCMM-2 was delivered via intravenous injection according to the specific treatment protocol depicted in Figure 7A. Following the injection, the fluorescence signal at the tumor site increased steadily, indicating the gradual accumulation of FCMM-2 in tumor tissue (Figure 7B). Quantitative analysis revealed that the strongest fluorescence intensity in tumor tissue was observed at 24 h post-injection (Figure 7D). For additional validation of the *in vivo* imaging findings, *ex vivo* imaging was carried out by retrieving major organs and tumors following treatment. Figure 7C, E showed that FCMM-2 was initially found in the liver at 12 hours post-injection, but by 24 hours its main presence had shifted to tumor tissue, indicating that the best time for ultrasound irradiation was 24 hours after administration. Following this, tumor volumes were measured in both primary and distant sites in order to evaluate the therapeutic impact of FCMM-2. Figure 7F, G, and S28 demonstrated that the tumor growth in the control and US-alone group continued to progress naturally, without significant restriction. In contrast, the FCM, FCM + US, MnO_2-x_ +US, and FCMM-2 groups displayed certain therapeutic effects on both primary and distant tumors through the synergistic efficiencies of ICD, cuproptosis, or STING pathway activation. For FCMM-2 + US group, the strongest antitumor effect was also observed, revealing the complete eradication of primary tumor and the outstanding suppression effect on distant tumor. Mouse body weights remained relatively stable throughout the study, as demonstrated in Figure 7H. Despite the control group mice starting to die around day 14, all mice in the FCMM-2 + US group survived beyond 50 days (Figure 7I), thus confirming the inhibitory effect of FCMM-2 on tumor recurrence. After undergoing treatment, mice from all treatment groups were euthanized, and their tumor tissues were gathered for H&E and TUNEL staining (Figure 7J, K). Both primary and distant tumors treated with FCMM-2 + US exhibited nearly complete necrosis based on staining results, demonstrating the excellent antitumor effectiveness of FCMM-2-mediated combination therapy.

Following the demonstration of the enhanced antitumor effectiveness of FCMM-2 in conjunction with SDT, CDT, cuproptosis, and cGAS-STING pathway activation, we proceeded to explore the immune response-related antitumor mechanisms *in vivo*. The FCMM-2 + US group had the highest CRT exposure among all groups (Figure S29), indicating that the heterojunction construction along with SDT and CDT could induce a stronger ICD effect. Furthermore, ROS staining of tumor in the FCMM-2 + US group exhibited a significantly higher ROS fluorescence signal intensity, which was higher than the other groups, demonstrating the highest production of reactive oxygen species within the tumor (Figure S30). These results suggest that by surface-modifying MnO_2-x_ on FCM, a significant enhancement in the synergistic effect of SDT and CDT was dissolved, resulting in the efficient inhibition of tumors growth.

**Figure 8:**
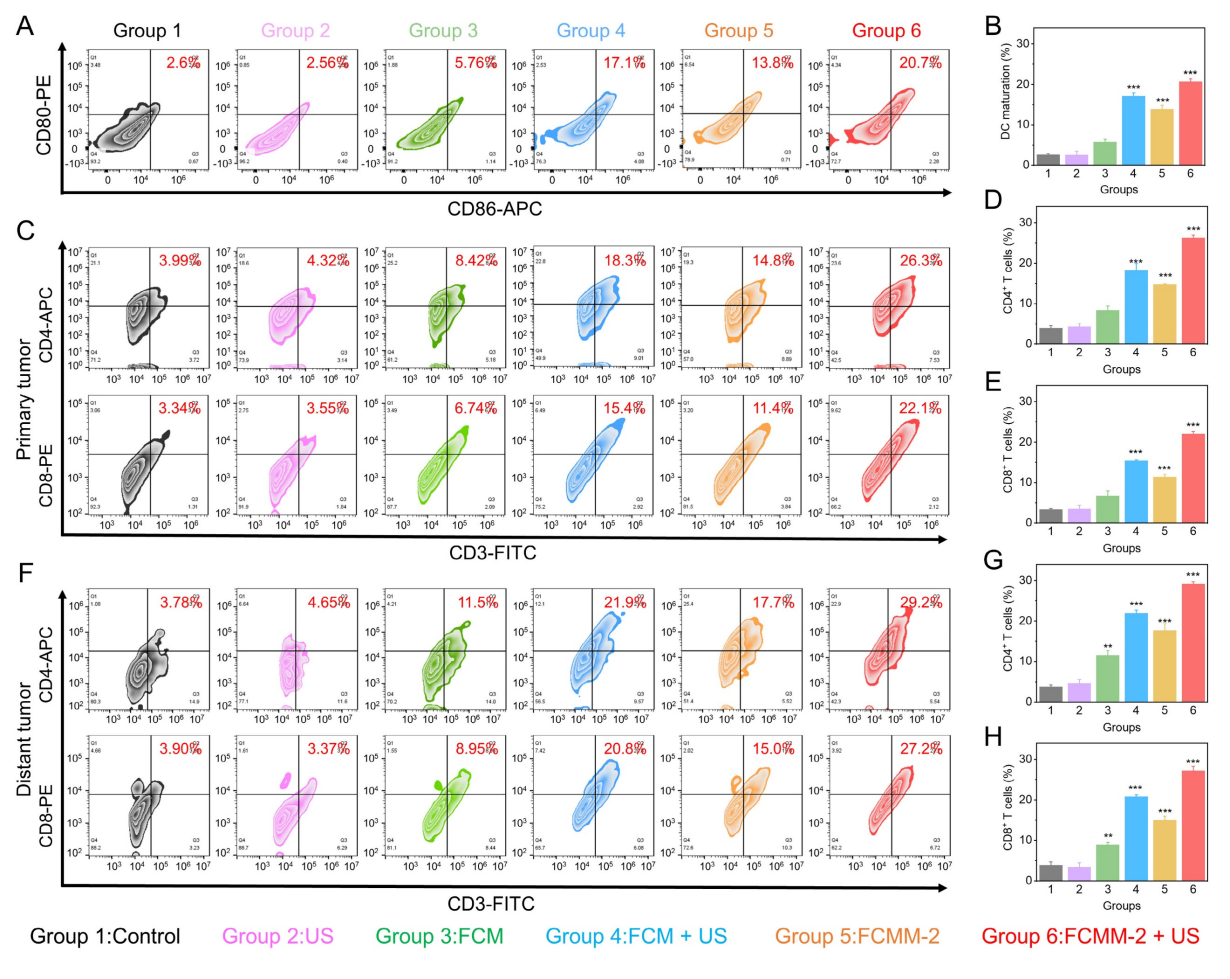
***In Vivo* Antitumor Mechanism of FCMM-2.** (A, B) DC maturation evaluation in tumor-associated lymph nodes in each group. (C-H) T cell activation level evaluation of primary (C-E) and distant tumors (F-H) in each group. Data are presented as the mean ± SD. (n = 3). **p < 0.01 and ***p < 0.001.

Upon the release of tumor antigens by tumor cells, they will prompt the maturation of dendritic cells 72, 73. The presence of mature DCs is facilitated the activation of antitumor T cells, and ultimately causing anti-tumor immune responses 74-76. Hence, we used flow cytometry to measure the changes in the ratio of DC cells, CD8^+^ and CD4^+^ T cells in the tumor tissues and spleens of mice in different treatment groups. As shown in Figure 8A, B, an increase was observed in mature DCs population expressing CD80 and CD86 in the FCMM-2 + US group, reaching 20.7%, compared with control group (2.6%). Therefore, CDT enhanced SDT therapy mediated by FCMM-2 could promote DC maturation effectively. After undergoing FCMM-2 + US treatment, a significant increase was observed in the precentages of CD8^+^CD3^+^ and CD4^+^CD3^+^ T cells in the primary tumors of mice, compared with that of the control group (Figure 8C-E). Subsequent to this, we examined the activation of T cells in spleen and distant tumors. Similarly, the spleen tissues and distal tumor tissues of the FCMM-2 + US group also showed the high expression of CD8⁺ T cells and CD4⁺ T cells, comparing to other groups (Figure 8F-H and S31). The result suggests that the activation of SDT, CDT, cuproptosis, and cGAS-STING could induce high-efficiency antitumor immune response.

**Figure 9:**
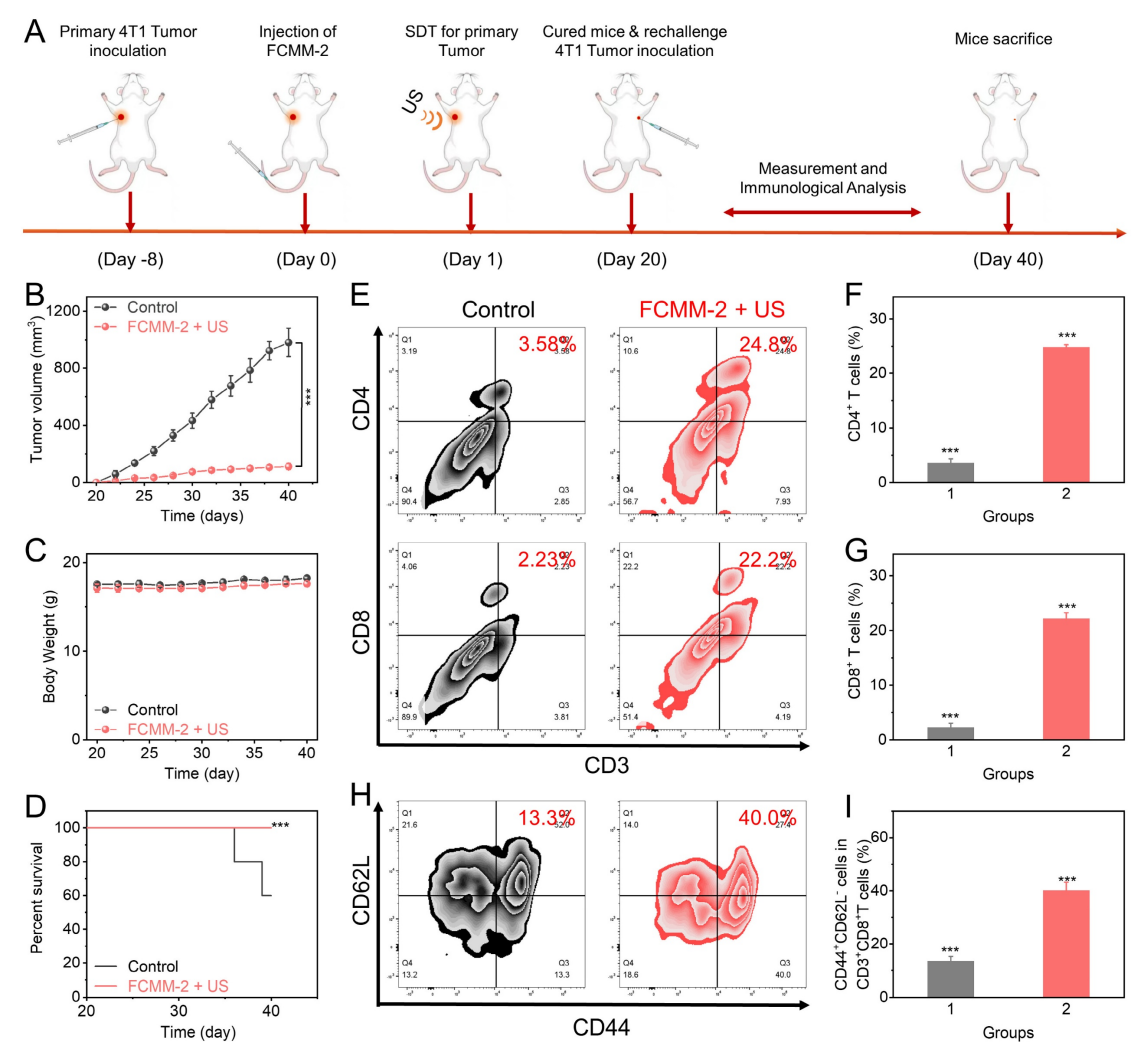
***In Vivo* Immune Responses of FCMM-2.** (A) The fabrication procedure of rechallenge tumor models and the administration of FCMM-2 + US treatment. (B-I) Determination of antitumor effectiveness and long-term immune memory response of FCMM-2-mediated combination therapy. Data are presented as the mean ± SD. (n = 5). ***p < 0.001.

To verify the immune memory effect of FCMM-2, we added a mouse re-challenge model. As shown in Figure 9A, a mouse unilateral tumor model was first established. After intravenous injection of FCMM-2 and US irradiation, the tumor in the mouse would be completely eradicated. On the 20th day, the same 4T1 tumor cells were re-inoculated on the other side of the cured mice, and five normal mice were inoculated with 4T1 tumor cells in the same area as the control group to observe the tumor growth conditions of the two groups of mice. As shown in Figure 9B, after the normal mice were inoculated with tumor cells, the tumors grew rapidly and reached about 800 mm^3^ after 20 days. However, for the mice cured by FCMM-2 + US, the growth of the re-inoculated tumor would be significantly inhibited, and their survival period would also be significantly prolonged (Figure 9C). To explore the potential mechanism of this phenomenon, after the mice were sacrificed, the tumors were taken out for immune analysis. As shown in Figure 9E-G, the flow cytometry detection of T cells showed that the contents of CD4^+^ and CD8^+^ T cells in the FCMM-2 + US group increased significantly. More importantly, the proportion of CD44^+^CD62Lˉ in these T cells was three times higher than that in the control group (Figure 9H, I). This cell subset is related to memory T cells, and the higher the content, the more memory T cells there are in the mouse body, which has a stronger immune memory function and can recognize and attack it after re-inoculating the same 4T1 tumor cells, achieving the purpose of inhibiting the growth of the re-challenged tumor. In conclusion, under the action of US, FCMM-2 not only can eliminate the primary tumor through a strong immune response but also can form a strong immune memory effect in the mouse body, thereby resisting the re-challenged tumor.

Finally, the assessment of biosafety for FCMM-2-mediated synergistic therapy was conducted. The H&E-stained images in Figure S32 revealed that no obvious abnormalities were found in the major organs, demonstrating the excellent biological safety of the FCMM-2-mediated synergistic therapy. The biochemical blood analysis and hematological index in Figure S33 showed that there were no significant abnormal blood indicators in the FCMM-2 group compared to the control group, suggesting the excellent biological safety of FCMM-2.

## Conclusion

In summary, we presented a TME-responsive nanoplatform by coating MnO_2-x_ onto FCM to create FCMM heterojunctions for the cascade amplification of antitumor immune response. By introducing Cu ions into Fe-MOF and then encapsulating a MnO_2-x_ shell, FCMM was developed as a high-efficiency sonozyme. This led to enhanced ROS production through heterojunction-mediated sonodynamic activity amplification, as well as multienzyme-mimic activities triggered by Fe/Cu/Mn ions, including CDT, GSH depletion, and alleviation of hypoxia. Endowing Fe-MOF with cuproptosis-triggering ability through doping Cu ions has not been previously reported. In addition, biodegradable heterojunctions based on Fe-Cu-MOF and MnO_2-x_ have not been previously reported. Through increasing the efficiency of ROS generation, immunosuppressive TME can be restructured, leading to potent ICD and facilitating DC maturation. Moreover, the activation of the cGAS-STING pathway and cuproptosis effect, triggered by the tumor-specific release of Mn^2+^ and copper ions, enhanced the maturation of dendritic cells. By employing a combination therapy involving CDT, SDT, cuproptosis, and cGAS-STING pathway activation via FCMM, primary tumors were entirely eliminated, and distant tumor growth was notably inhibited. Overall, this study demonstrated an effective strategy to integrate sonocatalytic therapy with immune regulation methods through ion engineering and heterojunction engineering, providing new insights into the design of multifunctional nanoplatforms for precise and durable antitumor therapy.

## Supplementary Material

Supplementary methods and figures.

## Data Availability

Data will be made available on request.
