# Supplementary material for: Bimetallic Cu/Fe-MOF-based heterojunction sonozymes for triple amplification of sono-immunotherapy through activating tumor-specific cuproptosis and cGAS-STING pathway
Source: Theranostics. 2026 Feb 26;16(9):4923–39. doi: 10.7150/thno.130381 (PMC12964376; doi:10.7150/thno.130381)
Supplement: Supplementary file 1 — Supplementary methods and figures. [file thnov16p4923s1.pdf]

Bimetallic Cu/Fe-MOF-based heterojunction sonozymes for triple amplification of sono-immunotherapy through activating tumor-specific cuproptosis and cGAS-STING pathway

Xueyuan Liu[a,1], Nan Wang[b,c,1], Jinming Cai[a,1], Jinyan Hu[a], Zhenlin Zhang[a], Chuanqi Feng[d,*], Dengyu Pan[a,*], Bijiang Geng[a,*]

[a] School of Environmental and Chemical Engineering, Shanghai University, Shanghai 200444, China

[b] Senior Department of Obstetrics and Gynecology, Chinese PLA General Hospital, Beijing 100007, China

[c] Department of Obstetrics and Gynecology, the First Medical Center, Chinese PLA General Hospital, Beijing 100853, China

[d] Shandong Provincial Key Laboratory of Monocrystalline Silicon Semiconductor Materials and Technology, College of Chemistry and Chemical Engineering, Dezhou University, Dezhou, Shandong 253023, China

[1] These authors contributed equally to this work.

* Corresponding authors.

E-mail addresses: chq_feng@dzu.edu.cn (C. Feng); dypan617@shu.edu.cn (D. Pan); bjgeng1992@shu.edu.cn (B. Geng)

Experimental Section

Synthesis of Fe-Cu-MOF (FCM)

FCM was synthesized via a solvothermal method. Briefly, 0.54 g of $FeCl_3 \cdot 6H_2O$ (4 mmol), 0.68 g of $CuCl_2 \cdot 2H_2O$ (2 mmol), and 0.498 g of terephthalic acid (H_2BDC, 2.5 mmol) were mixed with 60 mL of N, N-dimethylformamide (DMF). The mixture was sonicated and stirred for 30 min, then transferred into a Teflon-lined autoclave and heated at 100 °C for 12 h. After the reaction, the resulting product was washed several times with DMF and ethanol by centrifugation at 8000 rpm for 5 min each time. The final product was dissolved in water for further use.

Synthesis of Fe-Cu-MOF@MnO_{2-x}

Fe-Cu-MOF@MnO_{2-x} (FCMM) was synthesized by first dissolving 60 mg of FCM in 50 mL of deionized water. Subsequently, 20 mg of MnF_3 was added to the mixture. After sonication for 15 min, the solution was stirred at room temperature for 1 h. The resulting product was collected by centrifugation at 7000 rpm for 5 min and repeatedly washed with aqueous solution.

Similarly, three additional composites-denoted as FCMM-1, FCMM-2, and FCMM-3 were prepared by varying the mass ratio of FCM to MnF_3 to 1:1, 3:1, and 6:1, respectively, following the same procedure described above.

Synthesis of MnO_{2-x}

MnO_{2-x} was prepared by dispersing 40 mg of MnF_3 in 80 mL of deionized water. The mixture was sonicated and stirred at room temperature for 30 min. The resulting product was collected by centrifugation at 7000 rpm for 5 min and repeatedly washed with deionized water.

Characterization

The morphologies of FCM and FCMM were investigated by transmission electron microscopy

(TEM, JEM-2100F). UV-vis-NIR absorbance spectra were recorded using a Hitachi U3100 spectrophotometer. Fluorescence spectra were measured with a Hitachi F7000 spectrophotometer. Structural characterization including X-ray diffraction (XRD) and X-ray photoelectron spectroscopy (XPS) was performed using a Rigaku 18 kW D/max-2550 diffractometer and a Kratos Axis Ultra DLD X-ray photoelectron spectrometer, respectively. The hydrodynamic diameter and zeta potential were determined with a Malvern Nano ZS90 analyzer.

Sonodynamic performance measurement

Absorption spectroscopy and fluorescence spectroscopy were employed to determine the production of ROS under US irradiation (50 kHz, 1.0 W/cm^2) in the presence of FCM and FCMM-2. For the detection of $^1O_2$, a solution containing 60 μL of DPBF was added to 3 mL of solutions containing the same concentrations of MnO_{2-x}, FCM, and FCMM-2, respectively. After US stimulation, the absorbance change of DPBF at 418 nm was measured at 0, 2, 4, 6, 8, and 10 min to quantify $^1O_2$ generation. For the detection of $O_2^{-•}$, solutions of MnO_{2-x}, FCM, and FCMM-2 at identical concentrations were mixed with DHR123. After US irradiation for different durations, the emission spectra of the mixed solution were analyzed immediately following each irradiation period (Ex: 500 nm).

Measurement of •OH generation

The assay was performed by mixing $NaHCO_3$ buffer (25 mM) with GSH at varying concentrations (0, 0.5, 1.5, 5, or 10 mM) and the material (200 μg/mL). After 15 minutes of reaction, the mixture was centrifuged and the supernatant was collected. The collected supernatant was introduced into H_2O_2 solutions with different concentrations (0, 0.25, 0.5, 1, 1.5, or 2 mM), followed by the addition of 5μL of MB (10 mg/mL). The reaction was allowed to proceed at 37 °C for 30 minutes, after which the

absorbance at 650 nm was measured.

GSH peroxidase-mimetic activity

MnO_{2-x}, FCM, and FCMM-2 were separately mixed with 1 mM GSH in PBS solution. After different stirring durations, 300 μL of each mixture was combined with 2700 μL of PBS, followed by the addition of 4μL of DTNB (0.2 mM). The GSH depletion capability of MnO_{2-x}, FCM, and FCMM-2 was determined by measuring the change in the absorption peak at 410 nm.

CAT-like catalytic activity measurements

The O_2 generation capacity of MnO_{2-x}, FCM, and FCMM-2 was determined using a portable dissolved oxygen meter (JPBJ-608, Leici, China). Briefly, samples at identical concentrations (300 μg/mL) were mixed with H_2O_2 at varying concentrations (0, 0.1, 0.2, 0.3, and 0.4 mM). The dissolved oxygen concentration was measured, and the V_{max} was determined. The oxygen production capacity of the samples was also evaluated under varying pH conditions.

In vitro degradation tests

To evaluate the TME-response degradation behaviors of FCMM-2, the prepared samples (FCM, FCMM-2) were placed in buffer solution containing 5 mM GSH (pH 6.0) or PBS solution (pH 7.4). after storing different times, the absorption spectra of samples was measured. At 12 h, 48 h, or 72 h, FCMM-2 was taken out for TEM characterization, XPS, and XRD measurement.

MTT Assay

For the MTT assay, 4T1 and NIH-3T3 cells were obtained from the Cell Bank of the Chinese Academy of Sciences. To evaluate the cytotoxicity of MnO_{2-x}, FCM, and FCMM-2, NIH-3T3 or 4T1 cells were seeded in 96-well plates at a density of 5×10^3 cells per well. After cell attachment, the cells were treated with various concentrations of MnO_{2-x}, FCM, and FCMM-2 and co-incubated for 24 h.

US irradiation was applied 4 hours after the initial incubation of cells with the samples, followed by further incubation for 24 hours. Finally, cytotoxicity was assessed by standard MTT assay.

Live/dead cell staining

4T1 cells were seeded in 6-well plates and incubated for 24 hours. After incubation with the samples, the cells were subjected to different treatments according to the experimental protocol. Finally, cells from each well were collected and stained using a Calcein-AM/PI dual staining kit to distinguish live and dead cells. Fluorescent images were captured using a fluorescence microscope.

Detection of apoptosis

4T1 cells were seeded in 6-well plates and cultured at 37 °C for 24 h. The cells were then subjected to different treatments (Control, US, FCM, FCM + US, FCMM-2, FCMM-2 + US). Apoptosis was subsequently analyzed using an Annexin V-FITC/PI Apoptosis Detection Kit (Beyotime) by flow cytometry (Beckman, CytoFLEX LX).

In vitro ROS detection

For the in vitro detection of ROS, intracellular ROS levels in 4T1 cells after different treatments were measured using a ROS Assay Kit (Beyotime Biotechnology). Briefly, 4T1 cells were seeded into confocal dishes at a density of 1×10^5 cells per dish and cultured overnight. Following this, the dishes were subjected to respective treatments according to the experimental protocol (Control, US, FCM, FCM + US, FCMM-2, FCMM-2 + US). Subsequently, the cells were stained with DCFH-DA probe and DAPI. Fluorescence images were observed and captured using a fluorescence microscope.

Detection of mitochondrial membrane potential

4T1 cells were seeded into confocal dishes at a density of 1×10^5 cells per dish and cultured in an incubator for 24 hours. The cells were then subjected to six different treatment conditions (Control,

US, FCM, FCM + US, FCMM-2, FCMM-2 + US) and further incubated for 24 hours. Finally, the cells were stained using a JC-1 Mitochondrial Membrane Potential Assay Kit (Beyotime) and imaged with a fluorescence microscope.

Western Blot analysis

4T1 tumor cells were seeded in 6-well plates and incubated for 24 h. The cells were then treated with different samples (Control, US, FCM, FCM + US, FCMM-2, FCMM-2 + US). Following treatment, cell lysates were collected, and the protein concentration of each group was determined using the BCA method. Protein samples were separated with SDS polyacrylamide gel electrophoresis and blotted onto the PVDF membrane. The PVDF membrane was blocked with 5% non-fat dry milk for 1 h at room temperature, and incubated with anti-LIAS antibody, anti-FDX1 antibody, anti-TBK1 antibody, anti-IRF3 antibody, anti-STING antibody, anti-p-TBK1 antibody, anti- p-IRF3 antibody, anti- p-STING antibody, Tubulin antibody, or GADPH antibody overnight at 4 °C. after washing with TBST, then the PVDF membrane incubated with a diluted Goat-Anti-Rabbit IgG (HRP) for 2 h. Finally, the PVDF membrane was washed again and visualized with ECL reagent.

In vitro detection of ICD biomarkers

To assess CRT, HMGB1, and extracellular ATP levels, 4T1 cells were subjected to different treatments: Control, US, FCM, FCM + US, FCMM-2, and FCMM-2 + US. For CRT detection, cells were initially fixed with immunostaining fixative for 10 min, followed by blocking with immunostaining blocking buffer for 30 min. After washing, the cells were incubated with a primary CRT antibody at room temperature for 3 h. Following additional washes, the cells were treated with a secondary antibody (Alexa Fluor 647, Beyotime, China) for 1 h. After further washing, nuclei were stained with DAPI for 15 min. Finally, the cells were washed with PBS and imaged using a

fluorescence microscope. HMGB1 and ATP levels were measured according to the manufacturer's instructions using an HMGB1 ELISA Kit and an ATP Assay Kit, respectively.

In vitro assessment of DC maturation

Bone marrow-derived dendritic cells (BMDCs) were isolated from the femurs and tibiae of 6-week-old Balb/c mice and cultured for 7 days in RPMI-1640 medium supplemented with GM-CSF (20 ng/mL) and IL-4 (10 ng/mL). The BMDCs were then co-cultured with conditioned media from 4T1 cells treated with different groups (Control, US, FCM, FCM + US, FCMM-2, FCMM-2 + US) for 24 h. Subsequently, the cells were collected into suspension, stained with fluorescently labeled antibodies (FITC-anti-mouse CD11c, APC-anti-mouse CD86, PE-anti-mouse CD80, BioLegend, USA), and analyzed by flow cytometry.

Tumor model

Five-week-old female Balb/c mice were purchased from SLAC Laboratory Animal Co., Ltd. (Shanghai, China). All animal experiments were performed with the approval of the Institutional Animal Care and Use Committee of Shanghai University (Approval No. SYXK 2019-0020). To establish a bilateral tumor model, orthotopic tumors were first inoculated on the right flank. Seven days later, 4T1 cells were injected at the corresponding site on the left flank to generate a distant tumor model.

In vivo antitumor efficacy

Mice were randomly allocated into six groups (Control, US, FCM, FCM + US, FCMM-2, FCMM-2 + US) and received intravenous injections accordingly. After injection for 24 h, the tumors were irradiated with US (50 kHz, 1.0W/cm^2) for 10 min. The tumor volumes and body weights of each mice were recorded every 2 days. At day 4, tumors were harvested for H&E, ROS, and TUNEL staining.

The predetermined endpoints for all mice were defined as either natural death or attainment of a tumor volume of 1000 mm^3. For immune response analysis, primary and distant tumors, spleens, and lymph nodes were collected. The harvested tissues were processed into single-cell suspensions through enzymatic digestion, grinding, and filtration for subsequent evaluation of ICD markers, DC maturation, and T cell activation.

Statistical analysis

All experiments are performed with at least three independent replicates. Data are presented as the mean ± standard deviation (SD). Statistical significance between the experimental group and the control group is calculated with a two-tailed Student's t-test. * denotes a statistical significance (*$p < 0.05$, **$p < 0.01$, and ***$p < 0.001$) between the data of the experimental group and the control group.

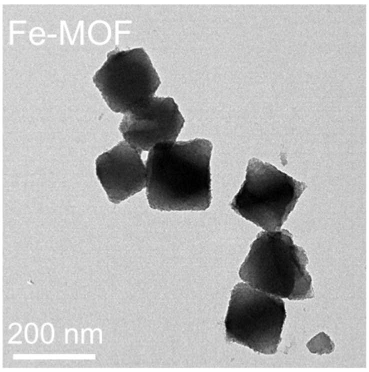

Figure S1. TEM images of Fe-MOF.

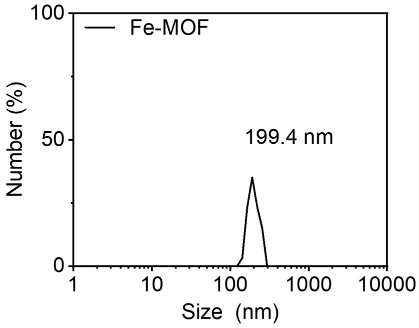

Figure S2. DLS measurements Fe-MOF.

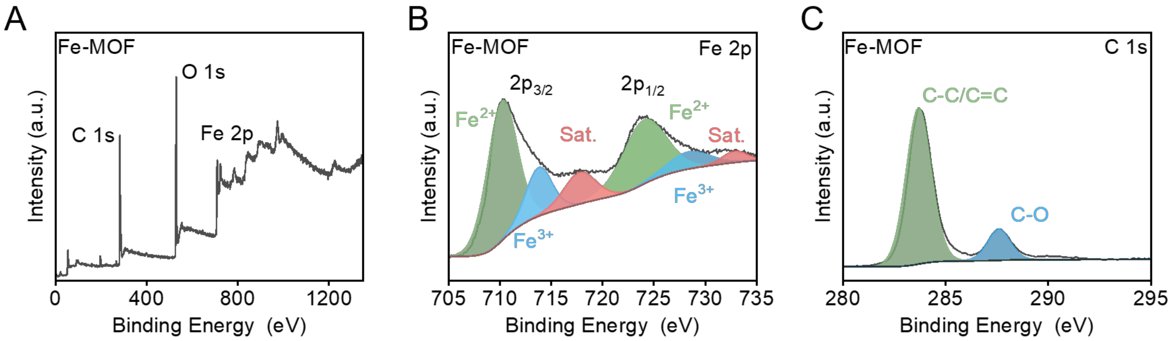

Figure S3. The survey XPS (A), Fe 2p (B), and C 1s (C) spectra of Fe-MOF.

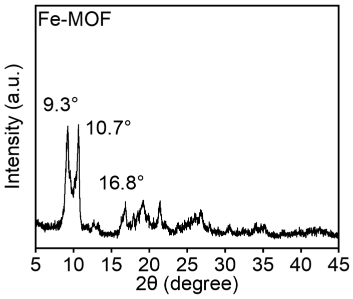

Figure S4. The XRD pattern of Fe-MOF.

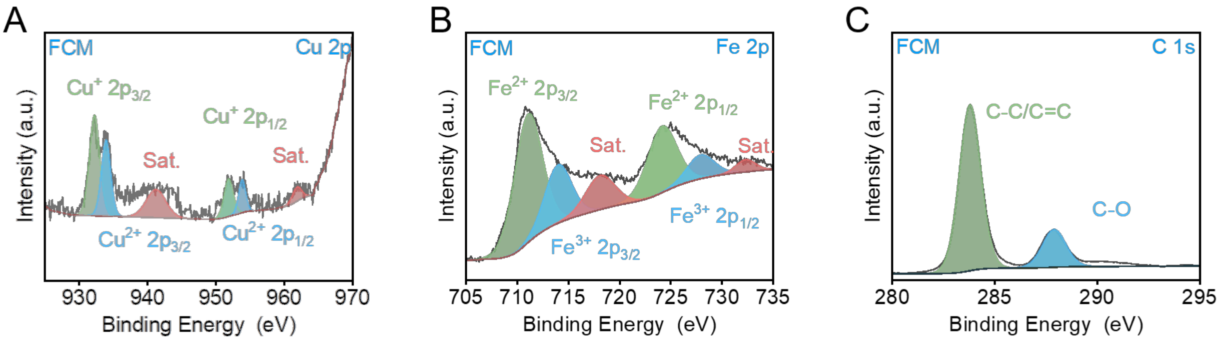

Figure S5. The high-resolution Cu 2p, Fe 2p, and C 1s spectra of FCM.

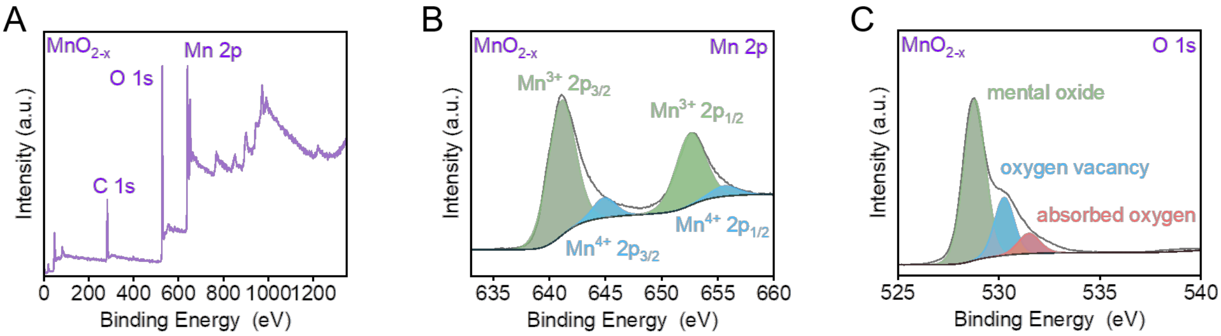

Figure S6. The survey XPS, high-resolution Mn 2p, and O 1s spectra of MnO_{2-x}.

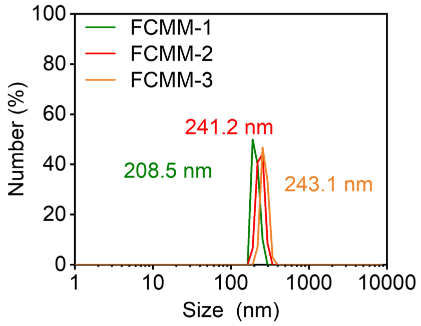

Figure S7. DLS measurements of FCMM-1, FCMM-2, and FCMM-3.

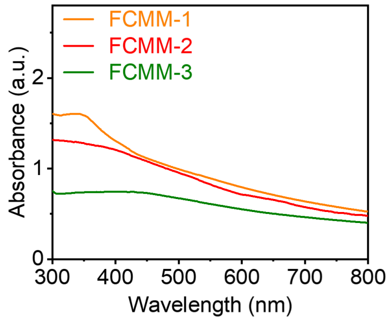

Figure S8. Absorption spectrum of FCMM-1, FCMM-2, and FCMM-3.

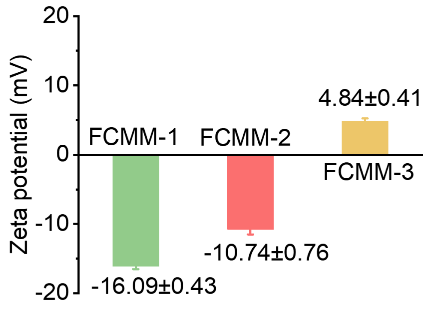

Figure S9. Zeta potential measurements of FCMM-1, FCMM-2, and FCMM-3. Data are presented as the mean ± SD. (n = 3).

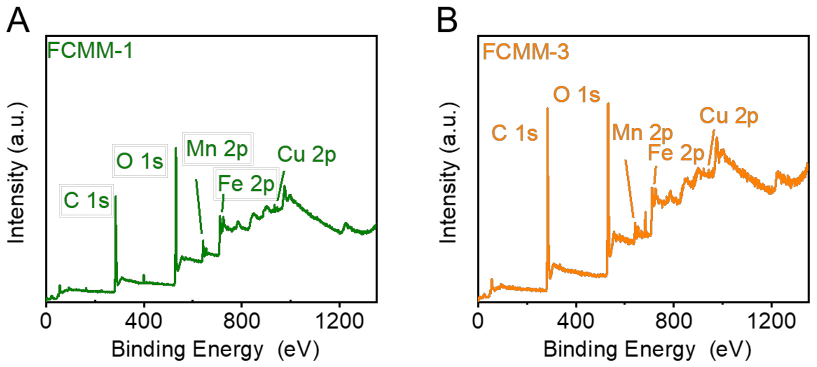

Figure S10. The survey XPS spectra of FCMM-1 and FCMM-3.

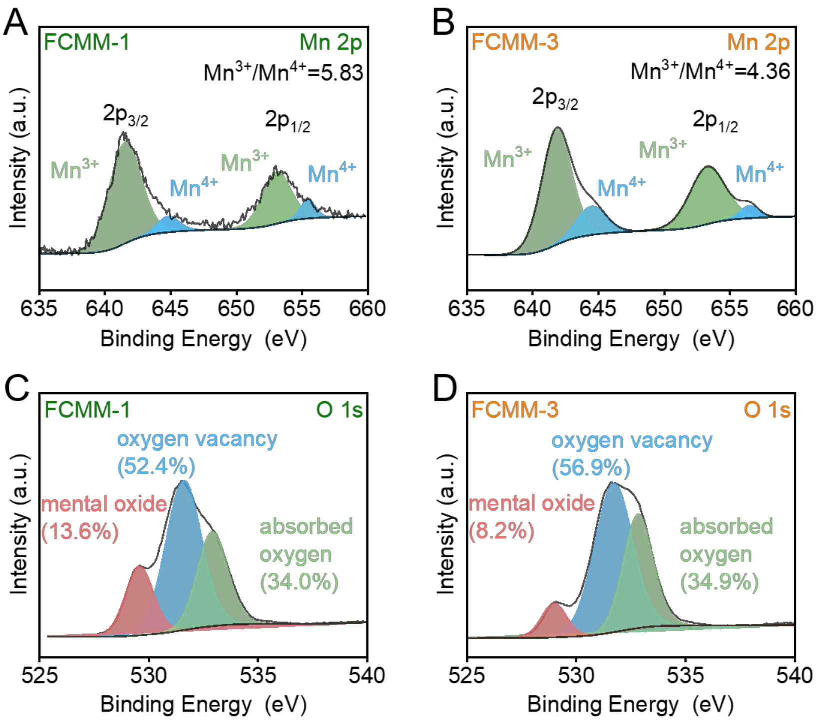

Figure S11. (A-D) High-resolution Mn 2p and O 1s spectra of FCMM-1 and FCMM-3.

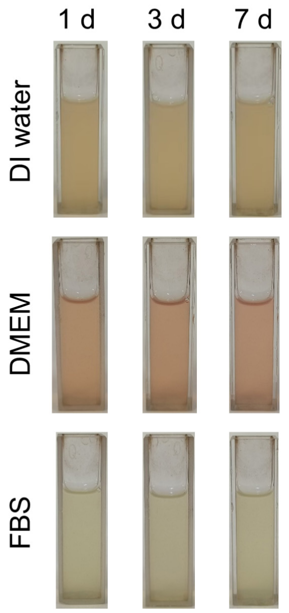

Figure S12. Photographs of FCMM-2 aqueous solution, DMEM solution, and FBS solution stored for different periods of time (1, 3, and 7 d).

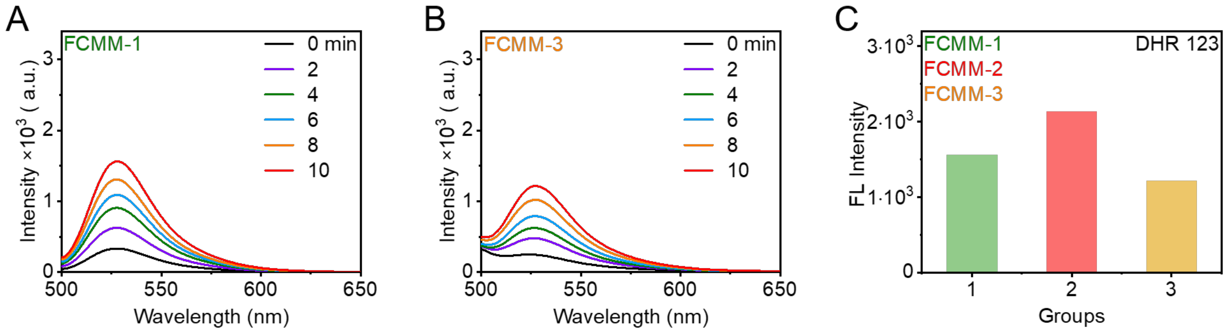

Figure S13. Comparison of the $O_2^{-\bullet}$ generation efficiency of FCMM-1, FCMM-2, and FCMM-3.

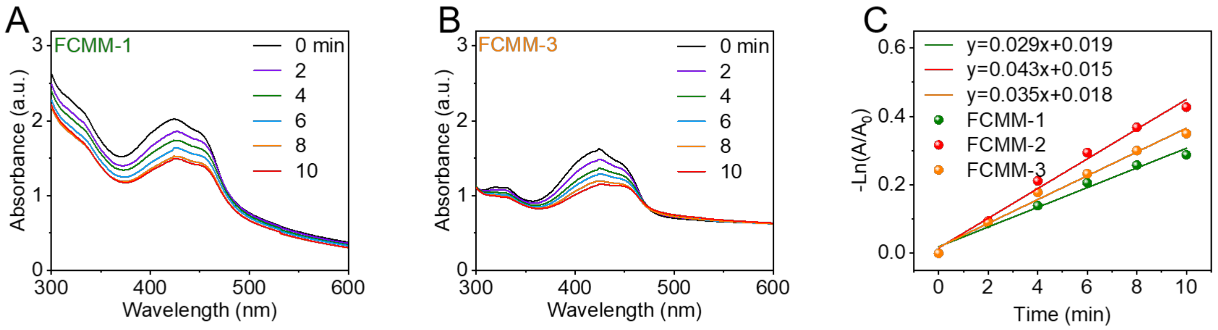

Figure S14. Comparison of the $^1O_2$ generation efficiency of FCMM-1, FCMM-2, and FCMM-3.

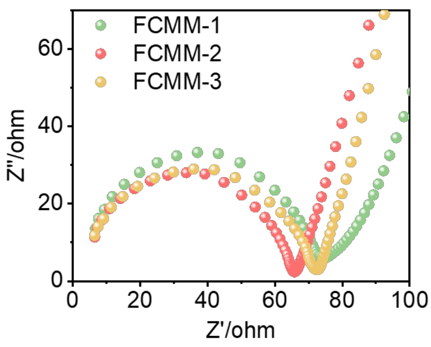

Figure S15. The impedance spectra of three different FCMM electrodes.

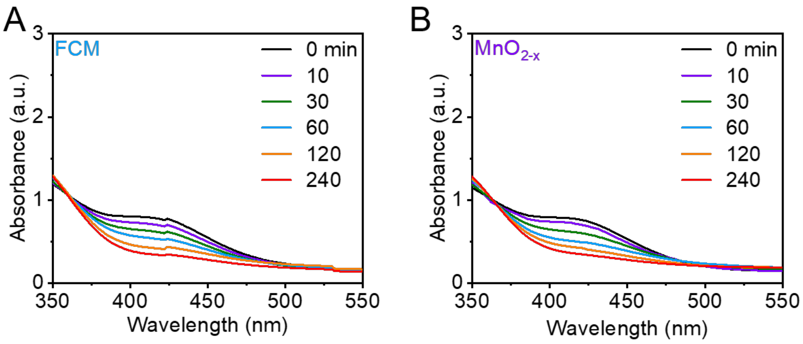

Figure S16. Absorption spectra measurements for evaluating the GSH consumption activity of FCM and MnO_{2-x}.

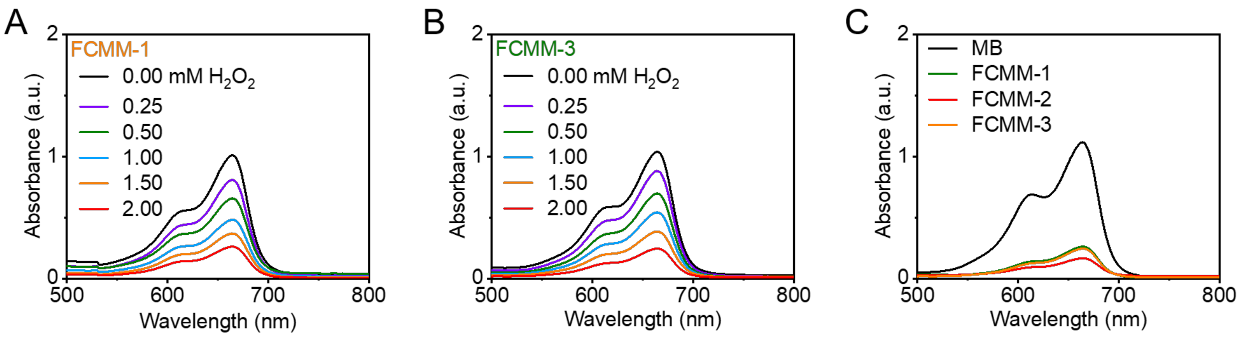

Figure S17. Absorption spectra measurements for the evaluation of the POD-like activity of FCMM-1, FCMM-2, and FCMM-3.

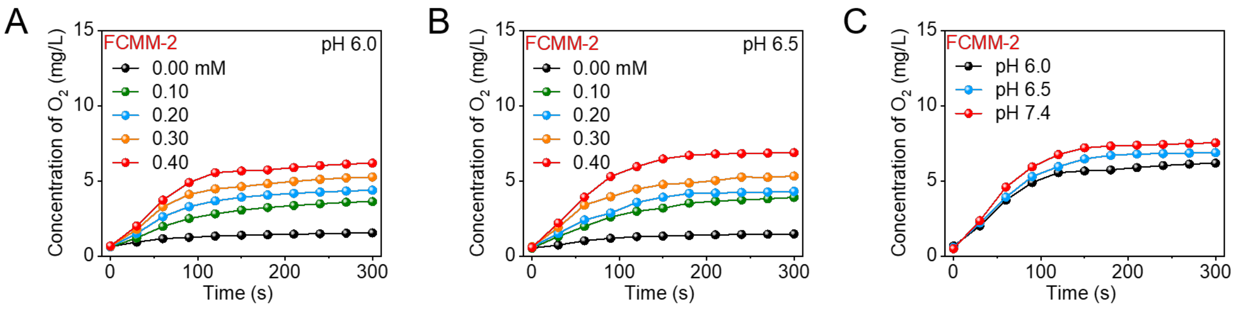

Figure S18. Comparison of the O_2 generation ability of FCMM-2 at different pH values.

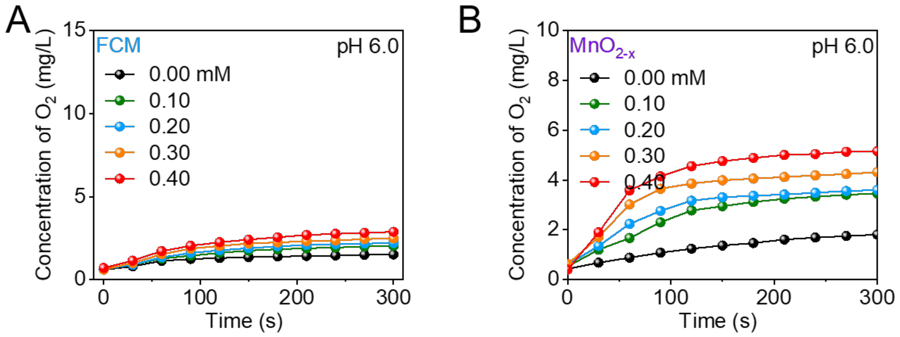

Figure S19. The O_2 generation ability of FCM and MnO_{2-x} at different H_2O_2 concentrations at pH 6.0.

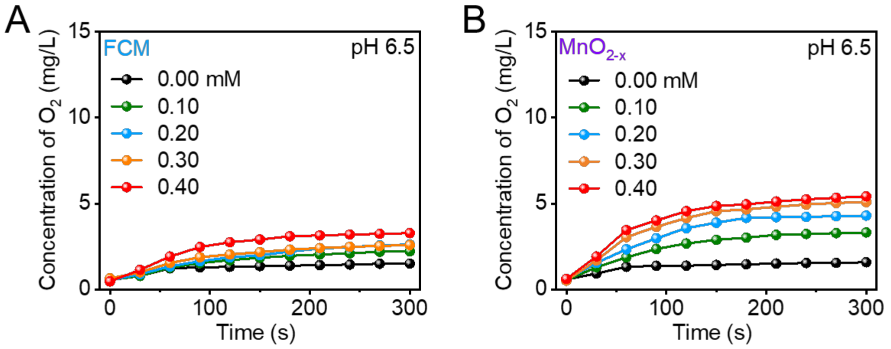

Figure S20. The O_2 generation ability of FCM and MnO_{2-x} at different H_2O_2 concentrations at pH 6.5.

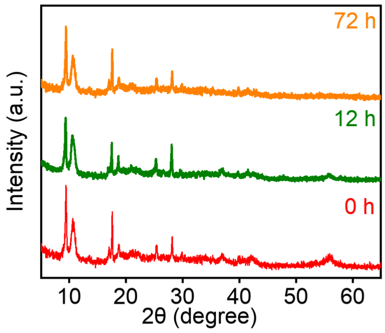

Figure S21. XRD pattern of FCMM-2 after degradation for 12 h and 72 h.

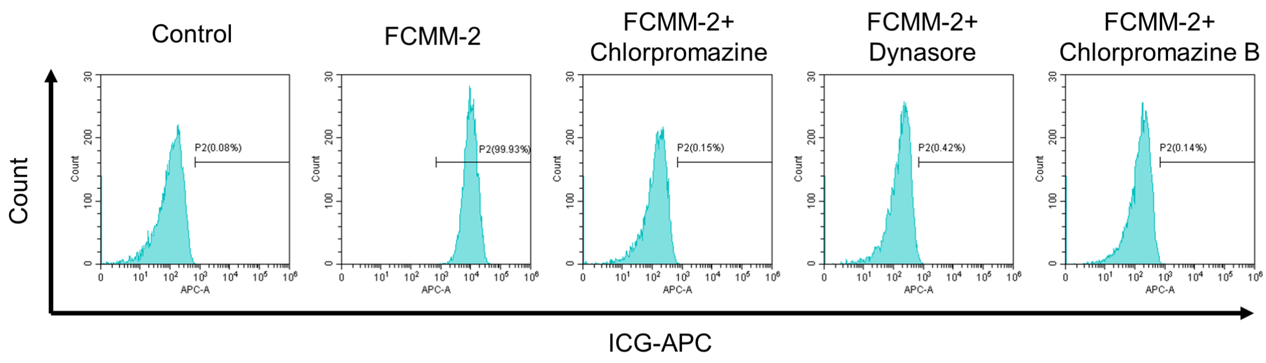

Figure S22. Cellular uptake of 4T1 cells after different treatments.

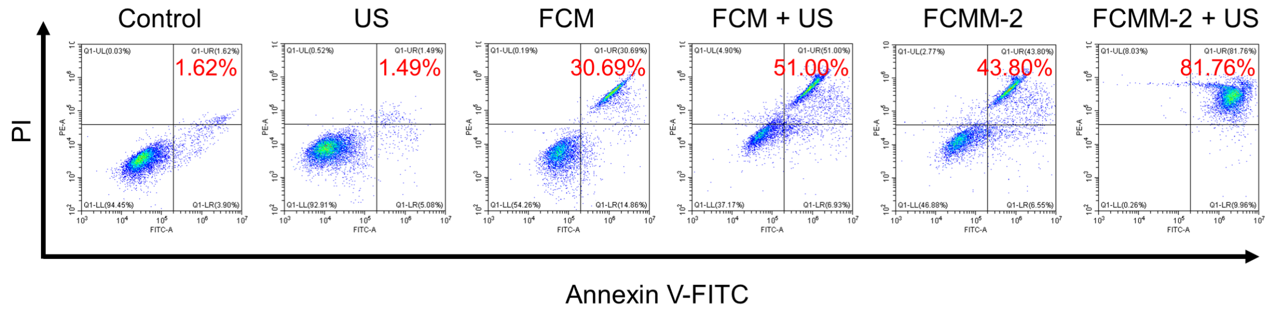

Figure S23. Apoptosis of 4T1 cells after different treatments.

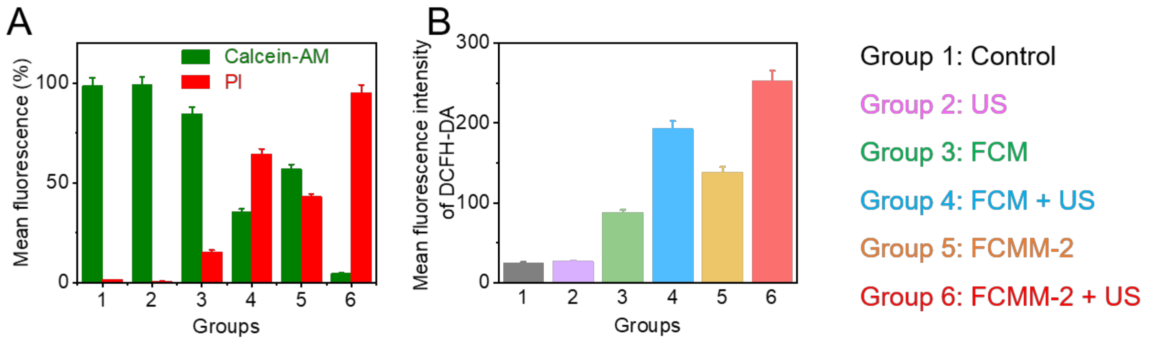

Figure S24. (A) Fluorescence quantitative analysis of live/dead staining of 4T1 (a) cells. (B) Fluorescence quantitative analysis of ROS staining of 4T1 cells. Data are presented as the mean ± SD. (n = 3).

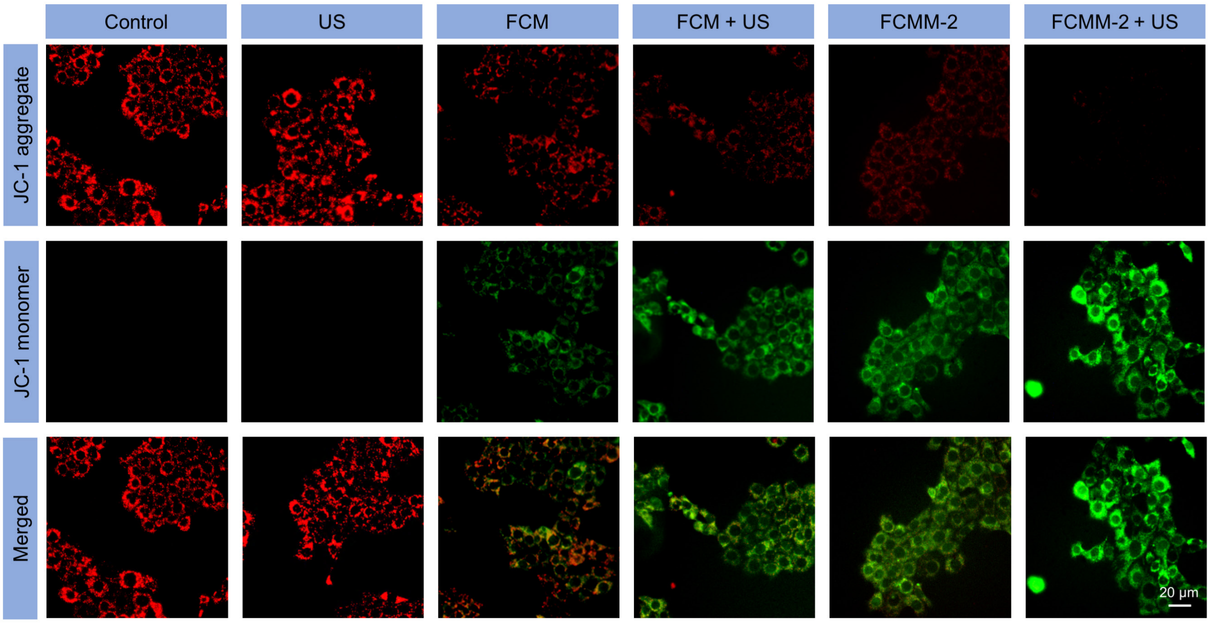

Figure S25. Mitochondrial damage staining images of 4T1 cells after different treatments.

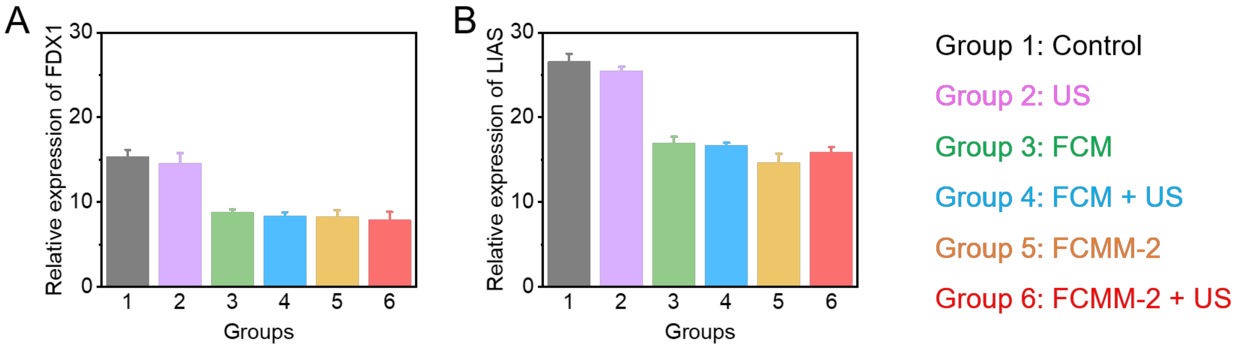

Figure S26. Expression levels of cuproptosis-related proteins in 4T1 cells after different treatments. Data are presented as the mean ± SD. (n = 3).

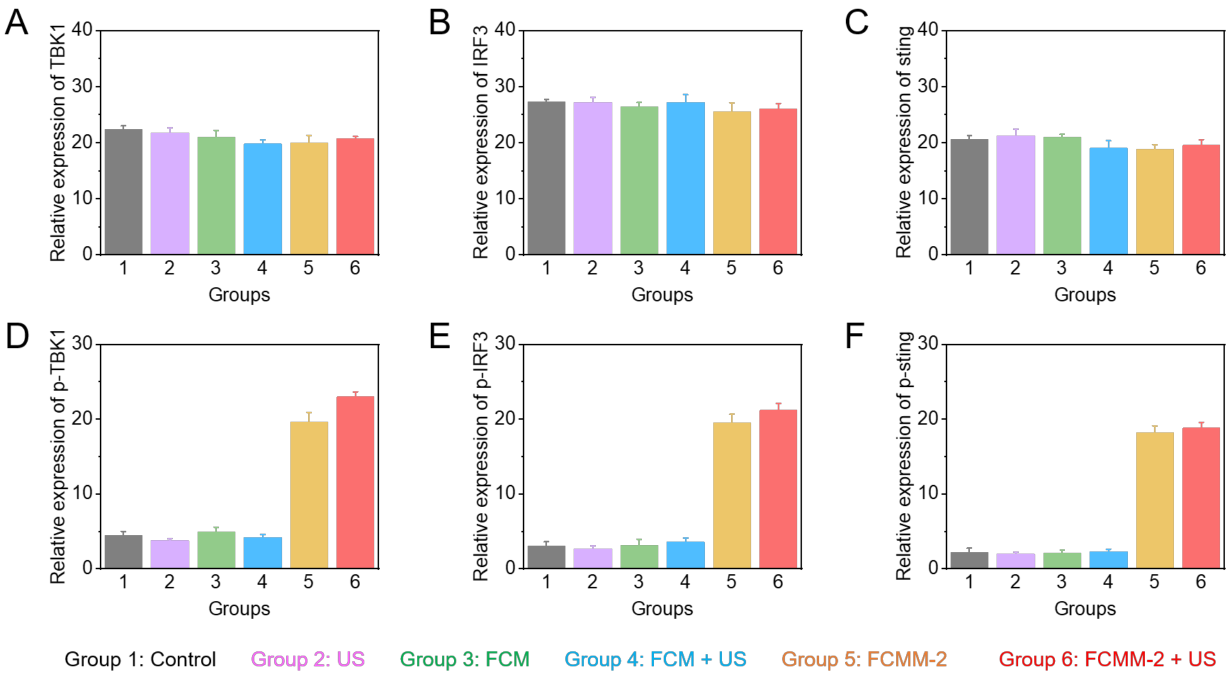

Figure S27. Expression levels of cGAS-STING-related pathway proteins in 4T1 cells after different treatments. Data are presented as the mean ± SD. (n = 3).

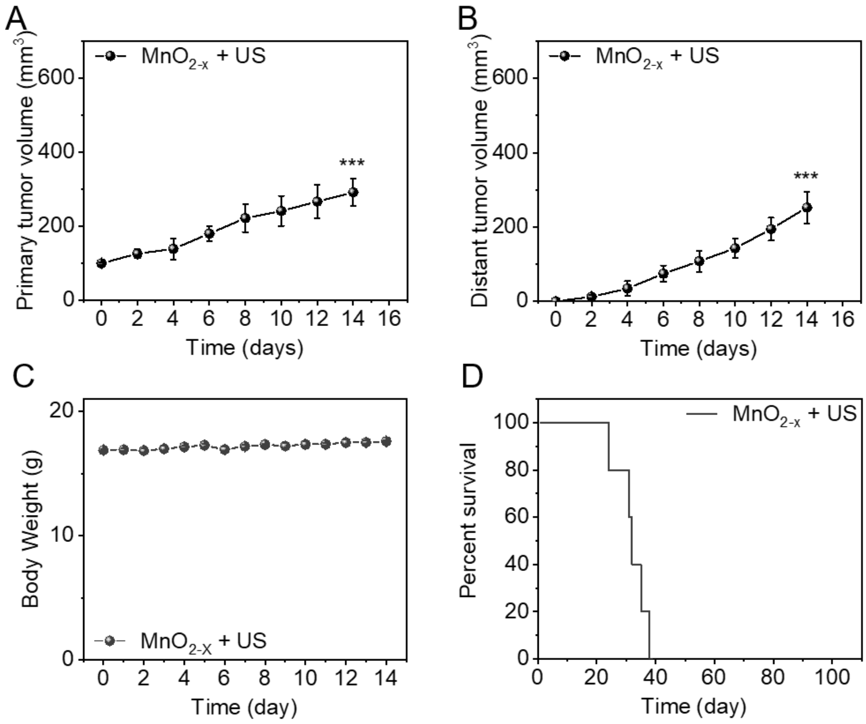

Figure S28. (A-D) Volumes of primary and distant tumors, body weight, and survival rate in mice after treatment of MnO_{2-x} + US. Data are presented as the mean ± SD. (n = 5). ***$p < 0.001$.

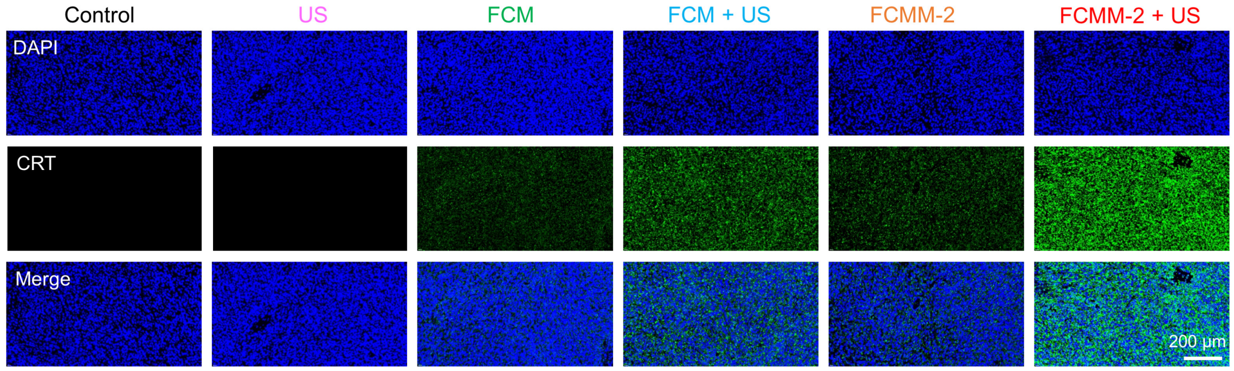

Figure S29. CRT level evaluation in tumor tissues after different treatments using immunofluorescent imaging.

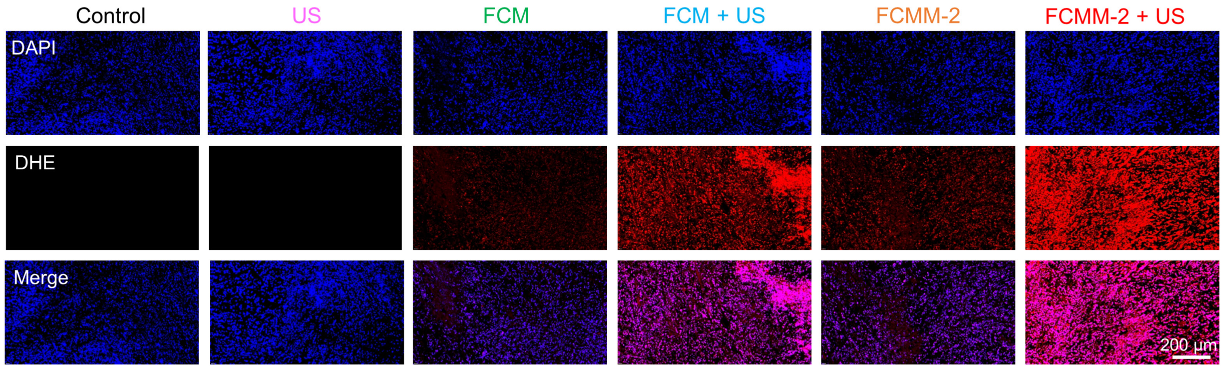

Figure S30. In vivo ROS staining of tumors in each group.

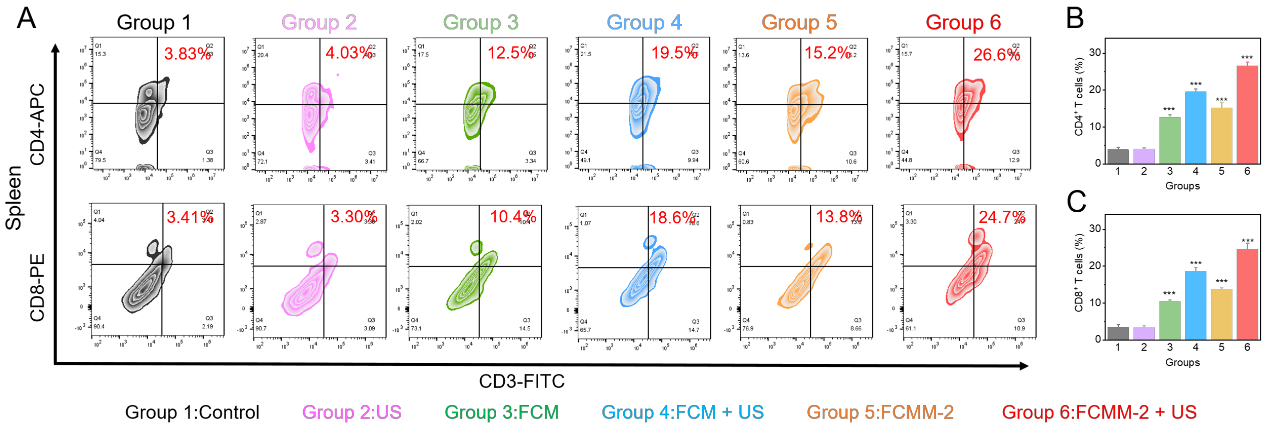

Figure S31. Evaluation of the expression levels of $CD4^+$ T cells and $CD8^+$ T cells in the spleen after different treatments. Data are presented as the mean ± SD. (n = 3). ***$p < 0.001$.

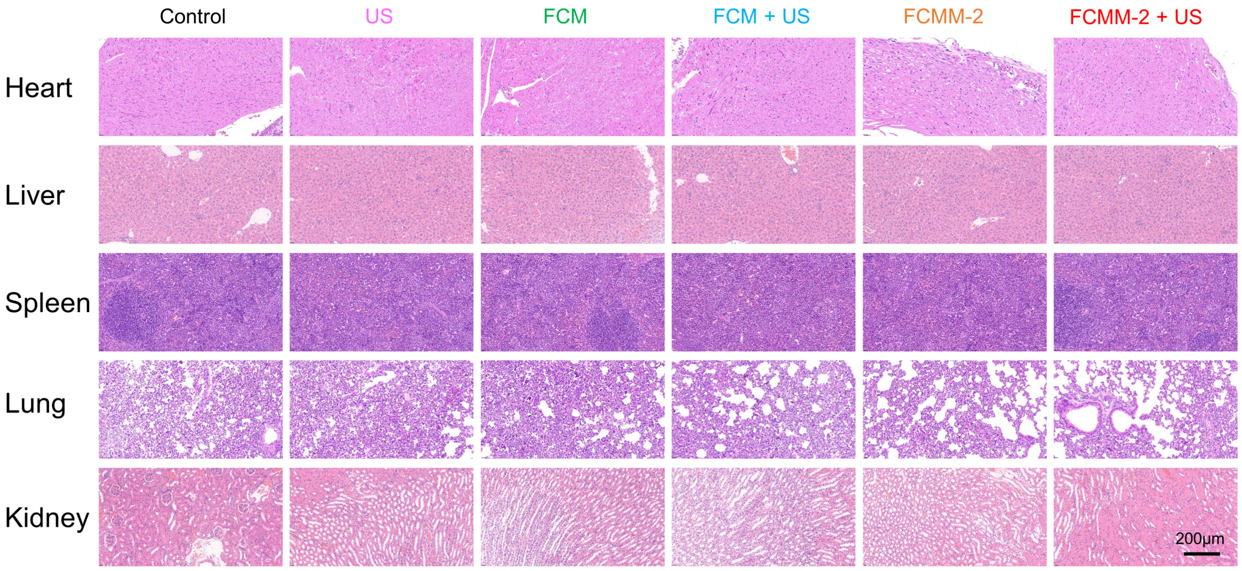

Figure S32. H&E staining images of the major organs in mice after different treatments.

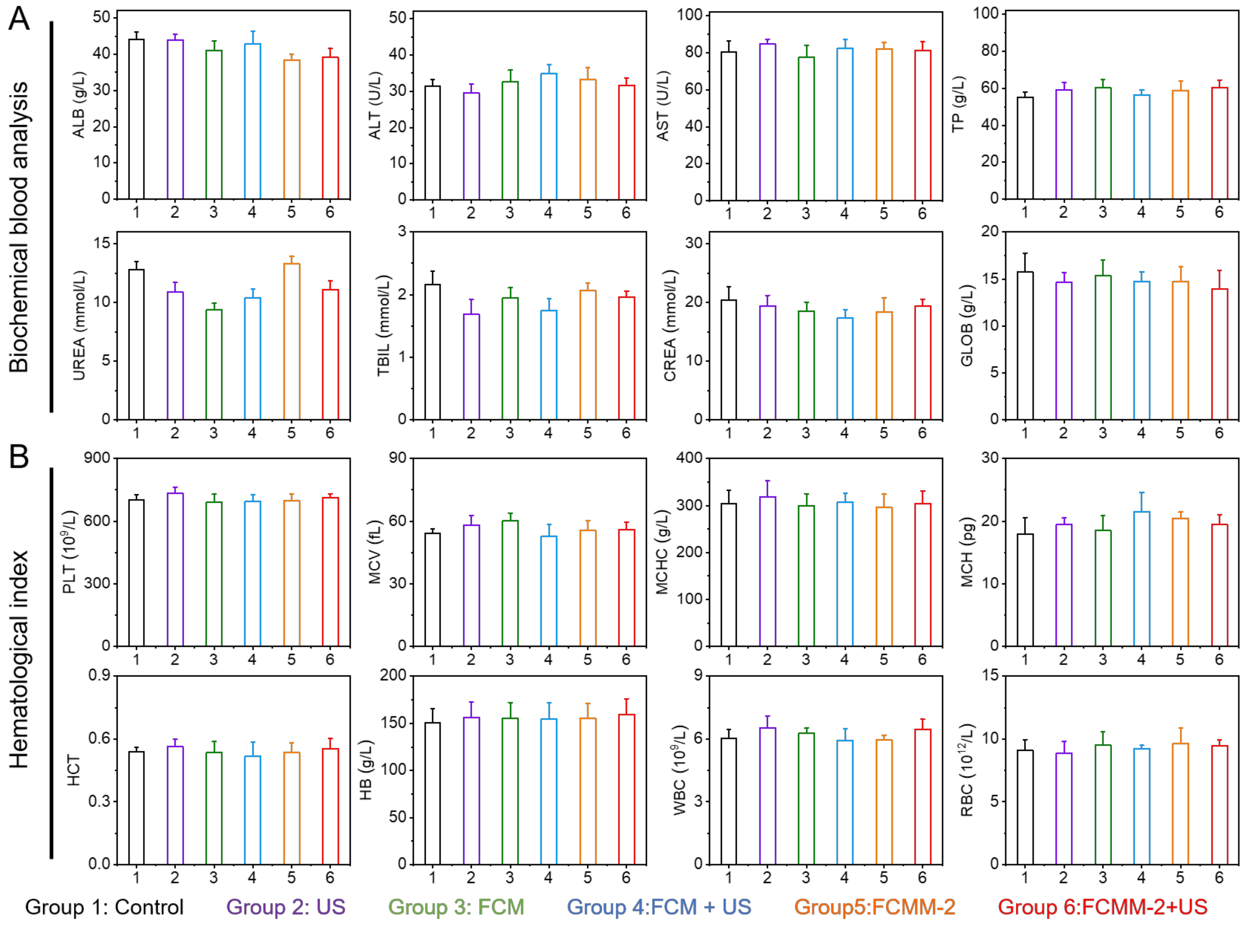

Figure S33. (A-B) Biochemical blood analysis (a) and hematological index (b) of the mice that were sacrificed at 14 days after different treatments. Data are presented as the mean ± SD. ($n = 5$). ***$p < 0.001$.
